# Repurposed Drugs and Plant-Derived Natural Products as Potential Host-Directed Therapeutic Candidates for Tuberculosis

**DOI:** 10.3390/biom14121497

**Published:** 2024-11-24

**Authors:** Rubhana Raqib, Protim Sarker

**Affiliations:** Immunobiology, Nutrition and Toxicology Unit, Nutrition Research Division, International Centre for Diarrhoeal Disease Research, Bangladesh (icddr,b), Dhaka 1212, Bangladesh; protim@icddrb.org

**Keywords:** phytochemicals, *Mycobacterium tuberculosis*, MDR, XDR, anti-TB drugs

## Abstract

Tuberculosis (TB) is one of the leading causes of death due to infectious disease. It is a treatable disease; however, conventional treatment requires a lengthy treatment regimen with severe side effects, resulting in poor compliance among TB patients. Intermittent drug use, the non-compliance of patients, and prescription errors, among other factors, have led to the emergence of multidrug-resistant TB, while the mismanagement of multidrug-resistant TB (MDR-TB) has eventually led to the development of extensively drug-resistant tuberculosis (XDR-TB). Thus, there is an urgent need for new drug development, but due to the enormous expenses and time required (up to 20 years) for new drug research and development, new therapeutic approaches to TB are required. Host-directed therapies (HDT) could be a most attractive strategy, as they target the host defense processes instead of the microbe and thereby may prevent the alarming rise of MDR- and XDR-TB. This paper reviews the progress in HDT for the treatment of TB using repurposed drugs which have been investigated in clinical trials (completed or ongoing) and plant-derived natural products that are in clinical or preclinical trial stages. Additionally, this review describes the existing challenges to the development and future research directions in the implementation of HDT.

## 1. Introduction

Tuberculosis, caused by infection with *Mycobacterium tuberculosis* (Mtb), is the oldest and deadliest infectious disease known to mankind, responsible for more than 10 million people inflicted with new active disease every year and 1.5 million deaths from tuberculosis (TB) in 2022 [1]. It is a potentially curable disease; the Bacillus Calmette–Guerin (BCG) vaccination and a combination of multiple antibiotics is typically used to prevent and treat the disease and control its spread. However, the BCG vaccine is not very effective, and standard treatment regimen for TB is lengthy, requiring about 6 to 9 months of therapy. The standard drug combinations (e.g., isoniazid, rifampicin, pyrazinamide, and ethambutol) have severe side effects, leading to decreased patient compliance with anti-TB treatment [2]. Moreover, epidemiological studies have shown that about one quarter to half of newly diagnosed TB patients also suffer from diabetes mellitus (DM) as a concomitant disease; in many countries, particularly in Sub-Saharan Africa, comorbidity with human immunodeficiency virus (HIV) infection/acquired immunodeficiency syndrome (AIDS) is very common [3,4].

Late diagnosis, non-compliance with treatment regimens by patients, incomplete dosage intake, and errors in drug selection by clinicians are some of the reasons that have led to the emergence of multidrug-resistant TB (MDR-TB) and extensively drug-resistant TB (XDR-TB) [4,5]. The global burden of MDR-TB is substantial: a staggering number of 440,000 new cases of MDR-TB occur annually around the world, causing an estimated 150,000 deaths. It is appraised that there are approximately 30,000 new cases of XDR-TB each year globally [6]. These strains are resistant to frontline antibiotics, making treatment more difficult and expensive and less effective, thereby conferring a huge burden on health systems. The rapid acquisition of resistance to the newly approved drugs for MDR TB, delamanid and bedaquili, is even more distressing. Thus, there is a pressing need to apply new therapeutic approaches, such as the development of shorter regimens that can swiftly reach the microbe in different tissue locations to eliminate it and curb the emergence or development of multidrug resistance. Conventional antimicrobial drug development and validation takes several years to decades; therefore, alternative strategies are of high priority.

In recent years, host-directed therapy (HDT) has emerged as an alternative therapeutic approach for infectious diseases. Instead of acting on the pathogen itself, HDTs strengthen the immune system to enhance its antimicrobial capacity and ability to identify the invading pathogen and neutralize it [7,8,9]. The effective application of HDT requires a comprehensive understanding of key host physiological processes involved during Mtb infection and the intricate interactions between mycobacterial and host defense factors, such as the diverse immune responses against Mtb infection, the immune-evasion mechanisms of Mtb, and the metabolic processes of immune and inflammatory cells.

## 2. Immune Responses Against Mtb

### 2.1. Innate and Adaptive Immunity

TB is spread through air when people with active pulmonary TB release tiny droplets carrying Mtb bacilli as a consequence of coughing, sneezing, or spiting. A nearby person can acquire an infection after inhaling the aerosolized droplets [10]. The first-line defense strategy is applied rapidly by the host in the lungs by employing alveolar macrophages, dendritic cells (DCs), and neutrophils [11]. These phagocytic cells engulf and degrade pathogens and present antigens after processing the degraded pathogen to help initiate an adaptive immune response. For an effective phagocytosis, pathogen recognition, engulfment, and phagosome maturation are essential. Macrophages and DCs recognize Mtb through pattern recognition receptors (PRRs) expressed on their cell surface; these stimulated PRRs activate transcription factors (e.g., nuclear factor kappa B (NF-κB)) through intracellular signalling to promote the expression of proinflammatory cytokines and chemokines. Moreover, macrophages produce host defense peptides, such as LL-37 and β-defensin-2 and -3, that exert microbicidal activity against a broad spectrum of pathogens including Mtb [12]. DCs use the receptor, dendritic cell-specific ICAM (intercellular adhesion molecules)-grabbing non-integrin (DC-SIGN), and toll-like receptor (TLR) for the recognition and self-activation of Mtb [13]. DCs secrete receptors for antigen presentation; they migrate to the lymph nodes to activate a Th1-type response and present these antigens to naïve T cells in the draining lymph node, bridging innate and adaptive immunity [14]. In response to the chemotactic signaling of cytokines and chemokines, there is massive influx of other inflammatory cells including neutrophils to the local infection site (lungs) to exert phagocytosis. Alveolar macrophages and recruited inflammatory cells organize themselves into compact structure called granuloma. Granuloma is formed in the lung to limit Mtb growth; however, in 90% of infected individuals, a small proportion of Mtb can survive, driving towards the caseation of granuloma with increased hypoxic necrotic centers, lipids, multinucleated giant cells, and epithelioid and foamy macrophages, surrounded by a rim of lymphocytes [15]. Mtb can remain inside the granuloma for a very long time in a dormant state, resulting in latent tuberculosis. At the early stage of infection and granuloma formation, neutrophils exert anti-TB effects by producing reactive oxygen species (ROS) to kill the phagocytosed mycobacteria and releasing azurophil granule proteins to help macrophages to control intracellular Mtb [16]. However, only a small proportion of neutrophils can kill internalized mycobacteria effectively; in the later stage, neutrophils become nonmotile and their excessive accumulation in the granuloma helps the pathogen to survive [11,17,18]. Natural killer (NK) cells secrete perforin, granzyme, and granulysin to kill Mtb-infected cells [19]; they also produce interferon-gamma (IFN-γ) and interleukin (IL)-22 and multiple signaling pathways to activate macrophages in order to inhibit the intracellular growth of Mtb. During TB infection, NK cells also seem to be involved in TB granuloma formation.

Adaptive immunity steps in at a mid-to-later phase of an infection, once the early response of innate immunity has initiated. Antigen-presenting cells (APCs), including macrophages and DCs, phagocytose Mtb, digest the pathogen, and load the antigens onto major histocompatibility complex (MHC) class I or II molecules, presenting antigens to CD4^+^T and CD8^+^T cells to initiate adaptive T cell responses [20]. The process of antigen presentation activates CD4^+^T cells, which in turn activate CD8^+^T cells [21]. Activated CD8^+^T cells together with NK and natural killer T (NKT) cells secrete various cytotoxic molecules (perforin, granzyme and granulysin) and cytokines for signaling to kill intracellular mycobacteria [22,23,24].

The role of humoral immunity in protection against Mtb infection is controversial; little evidence exists about protective role of antibodies against mycobacterial invasion. Only at the lung mucosal surface may antigen-specific neutralizing antibodies protect against bacterial invasion [25].

### 2.2. Death of Infected Phagocytes

After the phagocytosis of Mtb, macrophages eliminate Mtb contained inside the phagosome by fusing with a lysosome and killing by ROS inside the cell. Occasionally, Mtb can escape phagosomes into the cytosol [26]. Autophagy is a mechanism that can eliminate these cytosol-exposed Mtb; this process is mediated by intracellular signaling proteins, stimulator of interferon genes (STING), parkin, or Smurf1 (innate defense) [27,28]. Autophagy is a highly regulated non-inflammatory process where a cell digests and recycles the internal structures in its cytoplasm and degrades the internalized pathogens inside lysosomes. Apoptosis, a form of programmed cell death in macrophages, activates both innate and adaptive immunity and protects against TB. The necrotic death of phagocytes, however, induces inflammation in the neighboring cells and tissues, releases the Mtb bacilli in the local milieu, and exacerbates the disease [11]. These mechanisms of the death of macrophages are exploited by Mtb to escape the host defense and disseminate. The tuberculosis necrotizing toxin produced by Mtb inhibits apoptosis and promotes necroptosis in macrophages. Mtb infection also induces cell death in neutrophils. While dying, neutrophils release neutrophil extracellular traps containing bactericidal proteins that can capture Mtb [29].

### 2.3. Metabolism in Host Cells During Infection and Inflammation

Phagocytes and immune cells undergo metabolic changes during Mtb infection. Metabolic changes at cellular level are important for mounting appropriate immune responses against Mtb infection. Aerobic glycolysis increases in infected macrophages and drives towards M1 polarization, with the halting of the tricarboxylic acid cycle and accumulation of succinate. These events eventually lead to increased IFN-γ release, induce the production of inflammatory cytokines and host-protective eicosanoids, and finally the controlling of intracellular Mtb replication [30]. Eicosanoids from lipid metabolic pathways contribute to the regulation of the cytokine secretion from innate immune cells, influencing both inflammatory and anti-inflammatory responses and increasing apoptosis in infected macrophages. On the flip side, mitochondrial fatty acid oxidation in Mtb-infected macrophages is inhibited, and there is excessive accumulation of cholesterol and fatty acids in the cytoplasm, leading to the formation of foamy macrophages [31].

T cell metabolism is critical for the priming, and differentiation of T cells for rapid activation during an immune response. Lymphocyte activation and proliferation are regulated by three crucial factors: alterations in the expression of cell surface receptors, type of nutrient availability, and oxygen levels. For efficient activation of lymphocytes to counteract Mtb infection, the regulated production of cytokines, pyrimidine, glutathione, and mitochondrial ROS are needed. Both early and chronic Mtb infection dysregulate T cell glycolytic metabolism through several mechanisms [32].

## 3. Host-Directed Therapies in the Treatment of TB

HDT primarily enhances the antimicrobial activity of the host to control the infection, repairs the host defense processes that have been manipulated by the pathogen, and diminishes excessive inflammation to curtail tissue damage. HDT-based approaches are attractive for the treatment of infectious diseases because they are less prone to causing resistance in the pathogen. Diverse host factors come into play simultaneously and consecutively; thus, it becomes difficult for the pathogen to evade the different activated host defense mechanisms. Moreover, the host factors are evolutionarily conserved; therefore, a successful evasion will require substantial mutational changes in the pathogen which is not cost-effective for the microbe. Furthermore, adjunctive treatment with HDT may amplify the effectiveness of the mainstream antimycobacterial therapy for Mtb, since the mechanism of mycobacterial elimination by HDT is different from that of anti-TB drugs. There are various host pathways through which HDTs can hypothetically inhibit TB disease progression, such as modulating different immune cells, regulating cytokine signaling, and antimicrobial (e.g., host defense peptides and cytolytic molecules) and epigenetic processes [33,34]. Many mechanisms of HDT in the treatment for tuberculosis as adjuncts to standard anti-TB chemotherapy have been examined; few of these were also successful in increasing the effectiveness of conventional antibiotics. Some examples are (1) the suppression of host factors influencing immunopathogenesis and the different stages of Mtb infection; (2) the augmentation of innate and adaptive immune response-related features; (3) induce dampening factors to minimize inflammation and tissue damage; (4) inducing immune regulatory pathways; and (5) curbing function of bacterial products that aid in evading host defenses [35,36,37].

Globally, many HDT candidates are being evaluated and are at different stages of preclinical or clinical trials as adjunctive therapies. Among these drug candidates, some are repurposed/repositioned drugs licensed for other diseases, while some are plant-derived natural compounds [38]. Newer studies are focusing on the effects of HDT on comorbid conditions such as TB with DM and HIV-TB.

### 3.1. Repurposed Medicines

The repurposing of existing drugs that are licensed for the treatment of different diseases (such as cancer, diabetes, and cardiovascular diseases) provides significant advantages in treating drug-resistant TB. These medicines, when used as adjunct therapies to short-course chemotherapy regimens, can eliminate Mtb and prevent the development of drug resistance. A number of preclinical and clinical trials have used licensed drugs repurposed for the treatment of TB (Table 1); we have focused on phase 2I to 3 trials only (completed or under investigations), reporting various HDTs.

#### 3.1.1. Targeting Death of Infected Phagocytes

**Figure 1:**
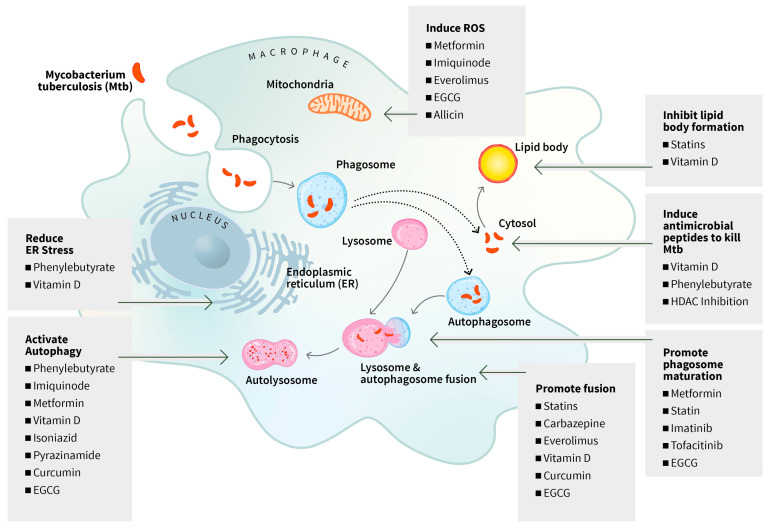
**Targeting death of infected phagocytes.** Macrophages engulf and phagocytose Mtb, which interferes with various processes to evade killing by the macrophage. (i) Some drugs such as metformin, everolimus, and allicin induce ROS production by mitochondria to enhance killing of Mtb. (ii) Statins, metformin, EGCG, carbazepine, and curcumin promote phagosome maturation or fusion of phagosome and lysosome. (iii) Phenylbutyrate, vitamin-D, metformin, and EGCG trigger autophagy, improve phagolysosomal fusion, and increase phagosome maturation and thereby enhance intracellular killing of the pathogen by macrophages. (iv) Once Mtb escapes from phagosomes into cytosol, some drugs such as phenylbutyrate and HDAC inhibitors induce production and release of antimicrobial peptides, e.g., LL-37, by the macrophages to kill the escaped microbe in the cytoplasm. (v) Within an infected macrophage, Mtb causes abnormal ER morphology and induces ER stress and subsequently apoptosis to promote intracellular survival. Phenylbutyrate and vitamin D can downregulate ER stress to prevent apoptosis. (vi) Mtb infection drives accumulation of neutral lipids and trigylcerides, with spontaneous lipid droplet formation leading to foamy macrophage development, which exerts inflammatory effects. Statin drugs and vitamin D can decrease blood cholesterol levels by blocking the synthesis of cholesterol and serve as a potential HDT candidate. EGCG: epigallocatechin gallate; ER: endoplasmic reticulum; HDAC: histone deacetylases; HDT: host-directed therapy; ROS: reactive oxygen species.

**Figure 2:**
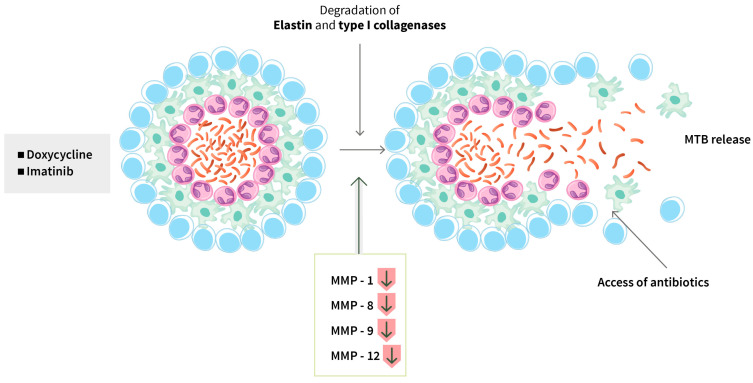
**Targeting granuloma degradation**. Granulomas are formed in the lungs to limit Mtb growth, though in majority of the infected individuals, a small percentage of Mtb may survive within the granulomas, driving towards caseation of granulomas and necrotic centers. Some drugs such as doxycycline and imatinib disintegrate granulomas’ structure by inhibiting MMP and blocking angiogenesis. This allows accessibility of antibiotics into the granulomas and infected cells and reduces the bacterial load. MMP: matrix metalloproteinase; Mtb: *Mycobacterium tuberculosis*.

Promote phagosome maturation: Mtb uses various immune evasion strategies through its virulence factors, and one of them is to inhibit phagosomal maturation. Therefore, accelerating phagosome maturation could be one approach for TB treatment. Imatinib is approved for the treatment of chronic myeloid leukemia (CML); it inhibits Abelson tyrosine kinase (Abl), known to affect lysosomal trafficking, and induces the expression and recruitment of the enzyme Vacuolar-type ATPase (v-ATPase), which is important for phagosomal acidification. Thus, imatinib promotes phagosomal acidification and improves Mtb killing within macrophages (Figure 1) [65]. In a murine *M. marinum* infection model, the use of imatinib as an HDT provided evidence of accelerating and regulating immune responses and limiting the lesion size and inflammation around granulomas [44]. A phase 2 trial is currently in progress in Vietnam and Nepal [44] (IMPACT-TB*; NCT03891901). Doxycycline and imatinib can disintegrate a granuloma’s structure by inhibiting matrix metalloproteinases (MMP) and blocking angiogenesis, thereby allowing antibiotics to enter into the granuloma and infected cells and reduce the bacterial load (Figure 2).

Autophagy induction and improving antimicrobial responses of macrophages: Mtb can escape the phagosomal membrane via its early secretory antigenic target 6 kDa (ESAT-6) secretion system-1 into the cytosol, where it replicates and induces the necrotic death of the infected cells and inflammation in neighboring cells and tissues. If autophagy is activated in an infected phagocyte, the cytosolic Mtb can be killed [66]. Autophagy induction thus can be one strategy of HDT for eliminating TB. Studies on the mechanism of action have revealed that many drugs and compounds can activate autophagy. A number of studies have reported a link between PRR and autophagy which can improve innate host defense against pathogens [67]. Imiquimod, an agonist of TLR7 (a PRR), is applied for treatment for superficial basal cell carcinoma [68]. This drug has been shown to trigger selective autophagy by increasing mitochondrial ROS and targeting autophagosomes to the mitochondria and nitric oxide (NO) production in mouse macrophages, thereby killing intracellular Mtb [69] (Figure 1). The active form of vitamin D blocks Mtb replication inside the infected macrophages through autophagic process [70]. Lipopolysaccharide in the cell wall of Mtb is recognized by TLR2 which triggers activation of vitamin D3-activating enzymes. Activated vitamin D3 induces the synthesis of an antimicrobial peptide, LL-37, which further enhances xenophagy [70]. Ex vivo studies have shown that phenylbutyrate, used for the treatment of various conditions, including urea cycle disorders, with or without vitamin D3 induces expression of LL-37, LL-37-dependent autophagy and intracellular killing of Mtb by macrophages, including multidrug-resistant Mtb [40,71,72,73]. Phenylbutyrate and vitamin D3 were applied as an adjunct HDT to standard short-course therapy in two clinical trials in pulmonary TB patients, including one that involved HIV-associated TB patients [39,41]. These studies showed that phenylbutyrate with or without vitamin D3 significantly improved clinical recovery, accompanied with increased expression of LL-37 and reduced intracellular Mtb growth in macrophages and earlier bacterial clearance from sputum (Figure 1).

The canonical anti-TB drugs isoniazid and pyrazinamide, which directly act on Mtb, can also activate autophagy in infected human macrophages, independent of their bactericidal activity [74,75]. Carbamazepine, an anticonvulsant medicine, activates Adenosine monophosphate (AMP)-activated protein kinase (AMPK) to induce autophagy during Mtb infection in human macrophages [76] (Figure 1).

#### 3.1.2. Modulating Immune Responses

**Figure 3:**
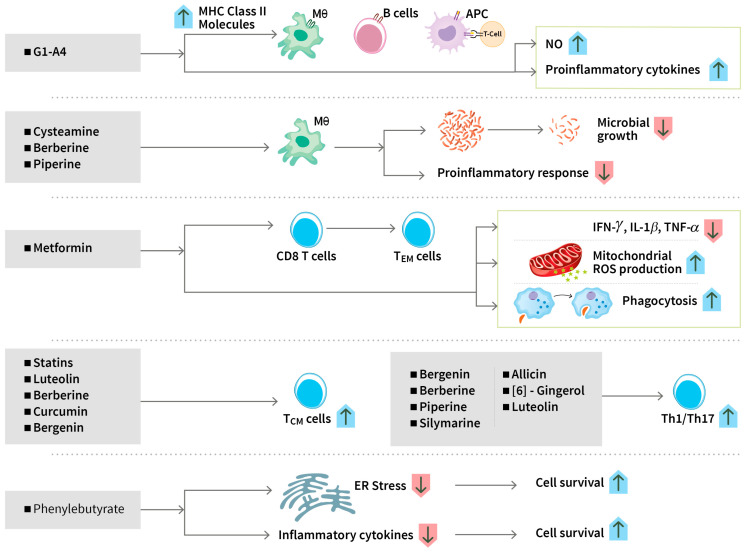
**Modulating cell-mediated immunity**. Both innate and adaptive immunity may be modulated by HDT candidate drugs. (i) G1-A4 enhances antigen-presenting capacity of macrophages, dendritic cells, and B cells and increases NO production and proinflammatory cytokine production. (ii) Cysteamine, berberine, and piperine reduce Mtb growth inside the infected macrophages. (iii) Metformin statins and some phytochemicals regulate cell-mediated immune responses by enhancing antigen-specific T-cell responses. Metformin enhances anti-mycobacterial responses by educating CD8+T-cells, reducing production of TNF-α, IFN-γ, and IL-1β, amplifying phagocytosis activity and mitochondrial ROS production of APCs. Statins, berberine, bergenin, curcumin, and luteolin can increase the proportion of central memory T cells. (iv) Phytochemicals such as piperine, silymarine, and allicin induce Th1/Th17 responses. (v) Phenylbutyrate reduces ER stress and inflammatory cytokine production and improves cell survival. APC: antigen-presenting cell; ER: endoplasmic reticulum; IFN-γ: interferon gamma; IL-1β: interleukin1 beta; MHC: major histocompatibility complex; NO: nitric oxide; ROS: reactive oxygen species; T_CM_: T central memory cell; T_EM_: T effector memory cell; Th: T helper cell; TNF-α: tumor necrosis factor alpha.

The modulation of immune responses by drugs as adjunctive HDT is an important approach to treating tuberculosis [7]. Endoplasmic reticulum (ER) stress plays a central role in innate immune signaling in response to microorganisms. Infection with Mtb mediates the overproduction of proinflammatory cytokines and induces ER stress in the host cells and promotes its intracellular survival [77]. Mycobacterium protein cell adhesion molecule M-cadherin (CdhM) can cause abnormal ER morphology and subsequently apoptosis through increasing the host stress markers binding immunoglobulin protein (BiP) and C/EBP Homologous Protein (CHOP) and the levels of X-box binding protein (XBP) 1 splicing and eukaryotic initiation factor-2α (eIF2α) phosphorylation to promote apoptosis [78]. Ex vivo experiments carried out using samples from a clinical trial showed that HDT with phenylbutyrate with or without vitamin D significantly reduced proinflammatory cytokines and chemokines and ER stress-related genes and induced autophagic markers in monocyte-derived macrophages [40]. Immunomodulatory compounds such as phenylbutyrate plus vitamin D inhibited the growth of clinical MDR tuberculosis strains in human macrophages via the induction of the antimicrobial peptide LL-37 and LC3-dependent autophagy. Vitamin D3 plus phenylbutyrate also had additive effects with isoniazid, showing potential therapeutic application in difficult-to-treat pulmonary TB [73] (Figure 1 and Figure 3).

In the context of adaptive immunity, many promising candidates have been investigated in in vitro studies; however, very few of these drugs have been tested as HDTs in clinical trials. Class II, major histocompatibility complex, transactivator (CIITA) is a major transregulator of MHC class II molecules; Mtb interferes with the antigen-presenting capacity of APCs by downregulating the expression of CIITA to prevent the production of MHC class II in macrophages critical for adaptive immunity [79]. G1-A4, a polysaccharide derived from *Tonispora cordifolia*, is a TLR4 agonist that upregulates MHC class II proteins and CD86 and increases the secretion of NO and proinflammatory cytokines, such as TNF-α, IL-6, IL-12, and IFN-γ, in a mouse model [80] (Figure 3). Cysteamine is a drug approved for the treatment of nephropathic cystinosis; it decreased the replication of Mtb strains in human macrophages [81]. Tamoxifen is a breast cancer drug. Even though it was reported to have a direct antibacterial effect on Mtb, it has a role as a repurposed drug in HDT in an experimental model for TB. The drug functions by promoting the delivery of mycobacteria to the lysosomes to engulf, process them into peptides, and transport them to the MHC-II loading compartment [82].

Metformin, the most widely used diabetes drug, has been used as a repurposed candidate adjunctive HDT for TB and the trials were associated with improved control of Mtb infection and decreased disease severity [83]. Metformin treatment exhibits a myriad of effects, such as reduced production of TNF-α, IFN-γ, and IL-1β, amplified phagocytosis activity and mitochondrial ROS production of APCs, and reduced chronic inflammation and lung pathology [84,85] (Figure 3). N-acetylcysteine (NAC) is a glutathione (GSH) precursor. In a phase 2 randomized clinical trial (RIPENACTB study), NAC used as an adjunct therapy during the first two months of anti-TB treatment was safe and also enabled mycobacterial clearance from sputum among HIV-associated TB patients [86] (NCT03281226, RIPENACTB). The NAC-treated patients showed markedly higher GSH and total antioxidant levels with a concomitant reduction in lipid peroxidation compared to the control group [56].

The mammalian target of rapamycin (mTOR) is a type of kinase protein which is switched on in certain types of cancer, allowing the cancer cells to grow and produce new blood vessels. mTOR inhibitors can stop the growth of these cancer cells and improve immune function [87]. Everolimus is a type of mTOR blocker. Auranofin is a disease-modifying antirheumatic drug that decreases pain during arthritis by decreasing inflammation. CC-11050 is a novel anti-inflammatory compound being used to treat diverse chronic inflammatory conditions and cytokine storms associated with infectious diseases. These three drugs and vitamin D3 were used in four separate arms in a phase 2 clinical trial; the findings showed that patients treated with everolimus and CC-11050 were safe and the treatment was reasonably well tolerated as an adjunctive HDT for TB, and also displayed enhanced recovery of lung function capacity [63] (NCT02968927). The modes of action of everolimus as an adjunct therapy are thought to be multiple. There is reduction of glycolytic activity which may mitigate inflammation in the lung and improve lung function; there may be increased ROS production in infected macrophages, inducing autophagy [88] (Figure 1).

#### 3.1.3. Modulating Anti-Inflammatory Responses

Mycobacterial infection can trigger a cascade of immune dysregulation. The host combats the infections by generating an inflammatory response that is crucial for controlling the pathogen, whereby a network of inflammatory cytokines, eicosanoids, prostaglandins, and other mediators are released. Uncontrolled and excessive release of these inflammatory mediators damage tissues and contributes to disease exacerbation [38]. Thus, balancing the inflammation could potentially optimize TB treatment.

**Figure 4:**
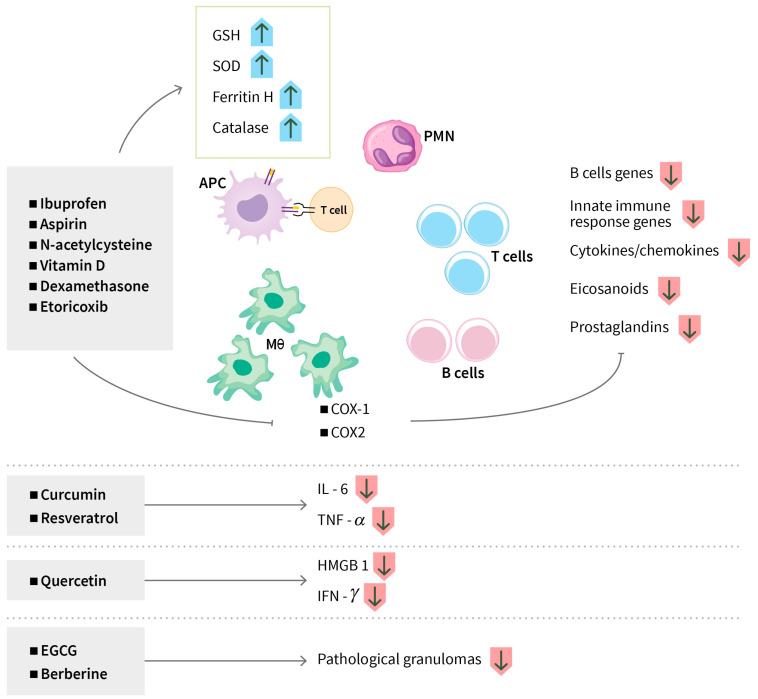
**Modulating inflammatory response**. (i) Non-steroidal anti-inflammatory drugs (NSAIDs), cyclooxygenase inhibitors (aspirin, ibuprofen, meloxicam, and etoricoxib), steroid drugs (dexamethasone), antioxidants (N-acetylcysteine), AMPK activators (metformin), and statins suppress proinflammatory responses, which decreases inflammation and tissue damage in infected macrophages. (ii) Phytochemicals such as curcumin, resveratrol, and quercetin reduce proinflammatory mediators. AMPK: adenosine monophosphate (AMP) -activated protein kinase; APC: antigen-presenting cell; COX: cyclooxygenase; EGCG: epigallocatechin gallate; GSH: glutathione; HMGB1: high mobility group box 1; IFN-g: interferon gamma; IL-6: interleukin 6; PMN: polymorphonuclear leukocytes; SOD: superoxide dismutase; TNF-a: tumor necrosis factor alpha.

Aspirins and ibuprofen are non-steroidal anti-inflammatory drugs (NSAID) that have analgesic, antipyretic (reducing fever) and anti-inflammatory effects. These drugs are inhibitors of cyclooxygenase-1 (COX-1) and cyclooxygenase-2 (COX-2), the enzymes involved in the synthesis of eicosanoids such as prostaglandins and leukotrienes that are biologically active lipid mediators. There is an increased synthesis of eicosanoids during leprosy and TB [89]. Prostaglandins have a significant role in the production of pain, inflammation, and fever (Figure 4). A number of clinical trials have been initiated to assess the safety and efficacy of NSAIDs (aspirin, ibuprofen, meloxicam, and etoricoxib) and steroid drugs (e.g., dexamethasone) as repurposed adjunctive HDTs of drug-sensitive and drug-resistant pulmonary TB as well as TB-meningitis, targeting the inhibition of COX enzyme activities [46,47,48,49,50,51,53] (NCT04575519; NCT02781909; NCT03092817; NCT02060006) (NCT04145258, NCT05917340) (Figure 4). One phase 2 trial in China utilized Salfasalazine as an adjunctive treatment of patients with pre-XDR-TB, which was found to be effective, safe, and well-tolerated [52] (ChiCTR2000032298).

Statins are primarily used for lowering blood cholesterol levels and reducing the risk of heart disease and stroke. In clinical trials, statins have been applied for their anti-inflammatory, antioxidative, anti-thrombotic, and immunomodulatory functions [59]. These drugs promote host defense mechanisms and inhibit pathological inflammation in infectious diseases. The mechanism of action includes the inhibition of formation of intermediates of the host mevalonate pathway, thereby compromising the immune evasion strategies of pathogens and their survival [90]. Atorvastatin given as a repositioned drug in HDT to uncomplicated, drug-susceptible pulmonary TB in Nigeria resulted in early sputum conversion and substantial decrease in chest X-ray severity scores after 8 weeks of treatment [91]. The mechanism of action of statins from experimental studies include anti-inflammatory effects, induction of autophagy, escalation of phagosome, and phagolysosome maturation [59]. Adjunctive rosuvastatin treatment was also safe in TB patients but did not produce substantive benefits on culture conversion [58]. A number of HDTs are ongoing using pravastatin as a repurposed medicine in South Africa (NCT03456102 and NCT03882177).

Doxycycline, a tetracycline antibiotic, has been shown to dampen host inflammatory responses by inhibiting MMP, which degrades collagen and other structural proteins to induce tissue damage and cavitation, down-regulate type I/II interferon and innate immune response genes, and up-regulate B cell biology-related genes. In a phase 2 trial in Singapore, doxycycline was given for 2 weeks as an adjunctive therapy that showed that the treatment was safe, and it reduced MMP-1, -8, -9, -12, and -13 expression in the sputum, suppressed type I collagen and elastin destruction, and reduced pulmonary cavity volume without altering sputum mycobacterial loads [54] (NCT02774993). A phase 2 clinical trial showed that doxycycline, when given in combination with anti-TB drugs, was well tolerated and the activity of type 1 collagenase and elastin was reduced in the sputum (NCT02774993). In a newly initiated phase 3 clinical trial in Singapore (Trial NCT05473520), 75 PTB patients in each arm will be given doxycycline or a placebo for two months with a follow-up of four months to determine improvement of lung function and decrease in tissue destruction [55].

#### 3.1.4. Regulating Metabolic Pathways

The accumulation of neutral lipids and trigylcerides during Mtb infection drives towards the necrosis of infected cells, facilitating the escape of Mtb into the cytosol. Hypercholesterolemia induces spontaneous lipid droplet formation and leads to foamy macrophage formation that exerts inflammatory effects through increasing inflammatory mediators. Excessive accumulation of lipid droplets in foamy macrophages can render them unable to control Mtb. Statins are used to decrease blood cholesterol levels by blocking the synthesis of cholesterol. Several statin drugs have been used as repurposed drugs in HDTs of TB, as described above.

Mounting evidence suggest that optimum activation of T lymphocytes for proliferation, clonal expansion, and execution of effector function is crucial to mount an effective immune response for the efficient clearance of infections. APCs activate T cells to undergo metabolic reprograming to support their effector functions [32]. Failure to activate T cells lead to T cell exhaustion. Mtb infection induces distinct defects to the metabolism of CD8^+^T cells and increases expression of both programmed death receptor 1 (PD-1) and other inhibitory receptor Cytotoxic T-lymphocyte-associated protein 4 (CTLA4) which is linked with decreased glucose uptake, glycolysis and mitochondrial respiration. Metformin increases the frequency of undifferentiated T cells, improves effector function and telomerase content [92]. Metformin enhances anti-mycobacterial responses by educating CD8^+^T-cells’ immunometabolic circuits [85] as described above. These actions of metformin can indirectly help improve the T cell-related metabolic pathways (Figure 3). The role of ibuprofen and aspirin as COX inhibitors that block eicosanoid synthesis have already been described above. Findings reflect that targeting the coupled metabolism of Mtb and the macrophage improves the control of infections [93].

### 3.2. Plant-Derived Natural Products (Phytochemicals)

**Figure 5:**
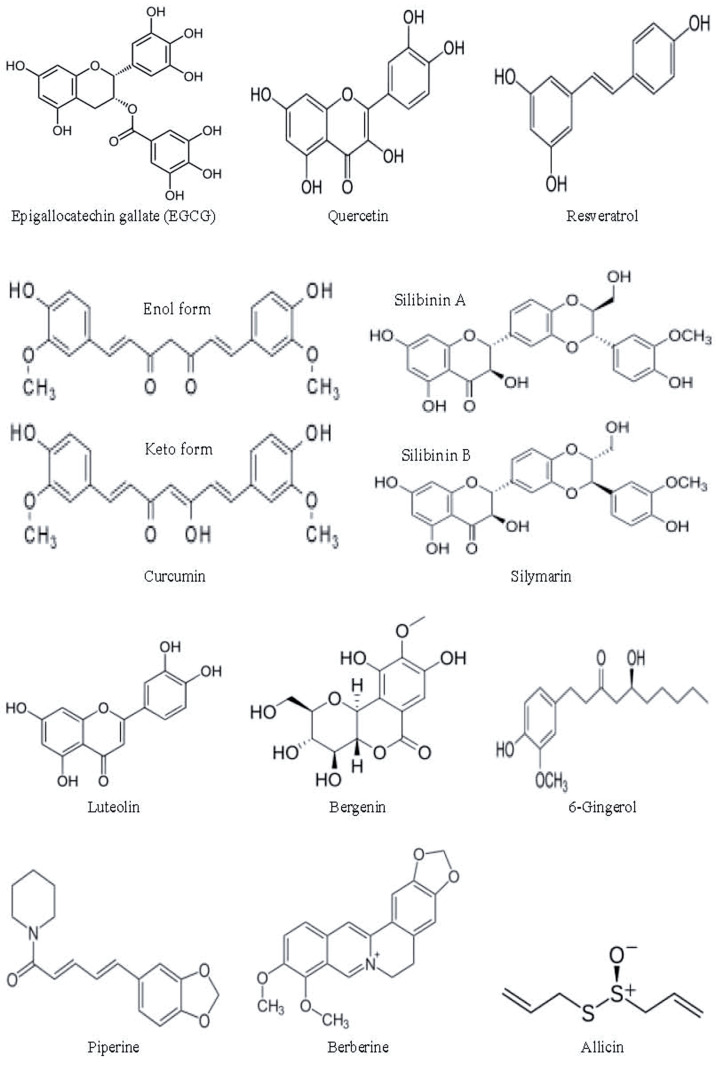
Chemical structures of the selected phytochemicals (Source: Wikipedia).

Historically, natural products have constituted a vital basis of diverse medicinal agents including anticancer, anti-inflammatory, antimicrobial, and antiviral drugs [94]. Plants are a source of a broad range of natural products having therapeutic properties against various diseases. Traditional ayurvedic treatments were based on the medicinal plants and plant-based products (primary and secondary metabolites), and knowledge of this traditional medicine system has provided the basis of exploring medicinal plants for manufacturing pharmaceutical products. The prospect of plant-derived natural products for drug discovery and phytopharmaceutical drug delivery systems has been reviewed extensively by Nasim N et al., 2022 [95]. In this review, we will highlight some of the phytochemicals that have undergone preclinical trials; a few of these products have also reached initial phases of clinical trials (Table 2). The chemical structures of the selected phytochemicals are given in Figure 5.

#### 3.2.1. Host-Directed Therapeutic Effects of Phytochemicals for TB

##### Targeting Cell Death of Phagocytes and Autophagy

Mtb can arrest phagosome maturation for its survival within macrophages by upregulating the host molecule tryptophan-aspartate containing coat protein (TACO). Epigallocatechin gallate (EGCG), one of the principal polyphenolic compounds of green tea was shown to downregulate the transcription of the TACO gene in human macrophages by inhibiting the Sp1 transcription factor, which was linked to inhibition of mycobacterium survival within macrophages [114]. Lysosome acidification by EGCG was also evident in mouse macrophages [115] (Figure 1). Supporting this finding, prospective population-based cohort and case-control studies showed a significant association between regular tea drinking and reduced risk of active TB [116,117]. Autophagy induction by microencapsulated EGCG was correlated with the dose- and time-dependent killing of TB bacteria inside mouse macrophages (Figure 1) [115].

After treatment with curcumin, a yellow-colored natural polyphenol compound isolated from turmeric (*Curcuma longa*), the co-localization of enhanced green fluorescent protein (GFP)-MTB H37Rv containing phagosome to lysosome was observed, suggesting enhanced phagosome lysosome fusion [101] (Figure 1). Curcumin also mediates intracellular clearance of drug-sensitive and MDR strains of Mtb by inducing apoptosis and autophagy in differentiated THP-1 human monocytes, primary human alveolar macrophages as well as in murine macrophage cell line RAW 264.7 [101,118]. These anti-MTB cellular functions of curcumin are mediated in part via the inhibition of NFκB activation [118]. In a mice model of pulmonary tuberculosis, treatment with curcumin was shown to decrease lung bacilli load [100]. However, in vivo studies are limited by poor bioavailability, poor gastrointestinal absorption, and rapid metabolism and elimination of curcumin. To overcome these limitations, a nano-formulation for curcumin (∼200 nm in size) was developed which enhanced the bioavailability by 5–7-fold in mice over regular curcumin [101,102]. A combination of curcumin nanoparticles and isoniazid led to a >99% decrease in the intracellular survival of MTB in macrophages [101]. The administration of curcumin nanoparticles along with isoniazid diminished the risk of tuberculosis reactivation and reinfection in mice, which is the major shortfall of the Directly Observed Treatment Short-course. Moreover, curcumin nanoparticles significantly shorten the duration of antibiotic treatment needed for complete clearance of Mtb from the lung, thereby reducing the possibility of generating drug-resistant Mtb strains [102]. The co-encapsulation of rifampicin and curcumin in polymeric nanoparticles was also shown to improve Mtb clearance from macrophages [119].

Resveratrol, a stilbenoid polyphenol found in more than 70 plant species including foods like peanuts, grapes, and cranberries, inhibited Mtb-induced apoptosis by activating Sirtuin 1 (Sirt1) in murine macrophages as well as in peripheral blood mononuclear cell (PBMC)-derived macrophages from healthy controls and patients with tuberculosis [120,121].

##### Modulating Immune Responses

The immune dysfunction of T cells induced by isoniazid is reversed by curcumin. Apoptosis of antigen-responding activated T cells is also prevented by curcumin through inhibition of the caspase-3 pathway activation. During primary infection in a mouse TB model, the co-administration of curcumin and isoniazid enhanced the total number of splenocytes, augmented the activation of both helper and cytotoxic T cells, and restored isoniazid-induced suppression in antigen-specific cytokine responses. Treatment with curcumin nanoparticles also protected the mice from reinfection by enhanced generation of central memory T cells [102] (Figure 3). Interestingly, by activating the host immune response, curcumin nanoparticles also enhanced the efficacy of the BCG vaccine [122].

Luteolin, a flavonoid found in many herbs, fruits, and vegetables, when co-administered with isoniazid in Mtb-infected mice, enhanced bacterial clearance, shortened the length of TB treatment, reduced the gross pathology of lung and spleen and prevented disease relapse. Luteolin or luteolin plus isoniazid also enhanced long-term anti-TB immunity by promoting central memory T cell responses in both spleen and lung [105]. Furthermore, luteolin enhanced the activities of natural killer and natural killer T cells and induced Th1 (IFN-γ and TNF-α) and Th17 (IL-17 and IL-22) cytokines [105] (Figure 3).

Bergenin, a secondary metabolic product (a polyphenol compound) found in different parts of several plants, stimulates Th1 and Th17 response and hinders the replication of Mtb in the lungs of Mtb-infected murine models. In infected macrophages, bergenin could activate the mitogen-activated protein kinases (MAPKs), extracellular signal-regulated kinase 1/2, and stress-activated protein kinase/c-Jun N-terminal kinases (JNKs) pathways, resulting in the production of TNF-α, NO, and IL-12 (Figure 3). Bergenin also shortens the duration of treatment and reduces immunological damage in mice receiving anti-TB treatment [106]. When co-administered with isoniazid, it increases the effectiveness of isoniazid in reducing bacterial load, including that of a multidrug-resistant Mtb strain, decreases isoniazid-mediated immune damage, and promotes the generation of long-lasting, antigen-specific central memory T-cell responses [107]. In a very recent study, bergenin has been shown to potentiate protective efficacy of BCG in mice by promoting a proinflammatory response milieu as well as mounting central and resident memory responses through modulation of the Akt-Foxo-Stat4 axis [123].

Berberine, an isoquinoline alkaloid obtained from the roots, bark, and rhizomes of medicinal plants, induces macrophage activation, Th1/Th17 polarization (Figure 3), enhancement of T effector memory (TEM), central memory (TCM), and tissue-resident memory (TRM) responses, and proinflammatory cytokine responses, resulting in enhanced host protection against both drug-sensitive and drug-resistant TB [111]. Analysis of human PBMCs derived from PPD+ healthy individuals revealed the modulation of the NOTCH3/PTEN/AKT/FOXO1 pathway as the central mechanism of berberin-mediated TEM and TRM responses. Enhancement of T cell memory by berberin lowered the risk of TB recurrence due to relapse and re-infection and also enhanced the efficacy of the BCG vaccine [111].

Piperine, an alkaloid isolated from the fruit of the plant Piper nigrum (black pepper), promoted the proliferation of T and B cells, enhanced Th-1 cytokines, and augmented macrophage activation in murine splenocytes. In Mtb-infected mice, piperine activated the differentiation of T cells into Th-1 phenotypes, as demonstrated by the secretion of elevated levels of Th1 cytokines (IFN-γ and IL-2) (Figure 3). A combination of piperine and rifampicin displayed a synergistic effect, resulting in the reduction of lung bacterial counts [124]. Risorine, a novel formulation of Rifampicin (200 mg) with bio-enhancer piperine (10 mg) and a standard dose of isoniazid (300 mg) was developed for a pilot study. In patients with drug-susceptible pulmonary tuberculosis, risorine, when given along with ethambutol and pyrazinamide, was found to be well tolerated and highly effective in sputum culture conversion [125]. Later, in a phase 3 clinical trial, the risorine group as compared to the standard WHO therapy group was found to maintain higher blood levels of rifampin, have a better safety profile, and demonstrate a higher sputum conversion rate at the end of 24 weeks, which was maintained until the end of the study [109].

Silymarin, a polyphenolic flavonoid extracted from milk thistle (*Sylibym marianum*) seeds, when administered with anti-TB drugs in a model of progressive pulmonary TB in mice infected with drug-sensitive or MDR Mtb strains, induced the expression of Th-1 cytokines IFN-γ, IL-12, and TNFα, producing significant therapeutic activity (Figure 3). Silymarin had a synergistic effect in reducing bacterial load and decreasing lung area affected by pneumonia [126].

6-Gingerol, a very potent pharmacologically active ingredient of ginger, inhibited the mycobacterial growth of dormant/starved bacilli and MDR/XDR strains inside the lungs, spleen, and liver of Mtb-infected mice. The reduction in bacterial load in the spleen was supplemented with the increased expression of proinflammatory cytokines and enhanced Th1/Th17 responses (Figure 3). Moreover, the anti-mycobacterial effect of isoniazid was enhanced by 6-Gingerol [108].

Allicin, an organosulfur compound and the main constituent of garlic, was shown to induce a protective Th1 response (Figure 3), resulting in a rapid reduction of mycobacterial burden in a mouse model of Mtb infection. The immune-dampening effects of anti-TB drugs was also reversed by the garlic extract [113].

##### Modulating Anti-Inflammatory Responses

The Mtb 19-kDa lipoprotein (P19), a component of the complex cell wall structure, induces an inflammatory response in human macrophages, which was shown to be attenuated by low concentrations of curcumin; curcumin inhibited the p38 MAPK pathway, decreasing the release of proinflammatory cytokines IL-6 and TNF-α [127] (Figure 4).

The Mtb-induced secretion of IL-6 and TNF-α in macrophages was reversed by resveratrol (found in grape skins, berries, and medicinal plants) (Figure 4). Resveratrol exerted this anti-inflammatory effect by augmenting Sirt1 expression, which in turn inhibited the activation of the transforming growth factor-β-activated kinase 1 (TAK1), MAPKs, and NF-κB pathways. Moreover, treatment with resveratrol reduced the susceptibility of mice to Mtb infection, as evidenced by lower bacterial loads and reduced histological impairment of the lungs [104].

In a rat model of TB, quercetin, a plant-derived polyphenol flavonoid inhibited inflammatory mediators, i.e., high mobility group box-1 (HMGB-1) and IFN-γ (Figure 4), and hindered activation of the NF-κB/TLR-4 axis.

Microencapsulated EGCG administered in a Mtb-infected mouse model by pulmonary delivery demonstrated a resolution of inflammation and significant reduction in bacterial load in the infected lungs; histopathological investigation revealed no or minimal pathological granulomas, lesions, and inflammation in the lungs [115] (Figure 4).

A berberin treatment of Mtb-infected mice along with isoniazid decreased the level of granulomatous inflammation in the lung (Figure 4) and had a synergistic effect on the anti-tubercular potential of isoniazid; the adjunct therapy was applicable to drug-sensitive as well as MDR and XDR strains [111]. Ozturk M et al. combined berberin with isoniazid and rifampicin in treating mice and found decreased lung pathology, which was associated with a reduction in the number of neutrophils, recruited interstitial macrophages, and CD11b+ dendritic cells. However, they did not find any additive or synergistic effects on bacterial burdens [112].

In Mtb-infected monocytes, the Mtb-induced expression of TNF-α mRNA was curbed by allicin in a dose-dependent manner; allicin also stimulated glutathione peroxidase activity, which correlated with reduction of ROS and TNF-α [128].

##### Hepatoprotective Role of Natural Products

The treatment of TB with isoniazid, rifampicin, and pyrazinamide are known to cause hepatotoxicity, which can vary from asymptomatic elevations of liver enzymes to liver failure.

Curcumin nanoparticles considerably attenuated isoniazid-induced hepatotoxicity and improved liver function in Mtb-infected mice by downregulating oxidative stress and inflammation. The hepato-protective effect of curcumin in mice as well as in liver cell line L-02 was correlated with a reversion of the SIRT1/peroxisome proliferator-activated receptor-γ coactivator (PGC)-1α/nuclear respiratory factor (NRF) 1 pathway, which was suppressed by isoniazid [103].

In mice treated with quercetin along with polyvinylpyrrolidone (QP), there was dramatic reduction of caseous necrosis caused by Mtb; adipose dystrophy of hepatocytes was observed in mice that did not receive QP treatment. When combined with the anti-tuberculosis medications isoniazid and streptomycin, the effect of QP was more pronounced; the spread of necrosis to other tissues was also prevented. This study suggests that quercetin and antibacterials have hepatoprotective effects against Mtb [129]. In a phase 2 clinical trial, the administration of QP as an adjunct to standard antimycobacterial therapy in patients with newly diagnosed pulmonary tuberculosis led to faster resolution of disease manifestation compared to patients with only standard therapy [97]. In a liver injury model in rat, pretreatment with quercetin improved isoniazid-induced histopathological changes in liver and substantially alleviated apoptotic cell death. Isoniazid-induced apoptosis in L02 cells was reversed by treatment with quercetin through the elevation of SIRT1 expression, increase of B-cell lymphoma (Bcl)-2 expression, and inhibition of the level of tumor suppressor P53, Bcl-2-associated X (Bax) protein, and cleaved-caspase 3 [130]. In another rat model, Sanjay S et al. showed that the protective effect of quercetin on liver injury was mediated by the activation of nuclear factor erythroid 2-related factor 2 (NRF2) and consequential suppression of oxidative stress and boosting of endogenous antioxidant levels [131]. Isoniazid-induced hepatotoxicity was also alleviated by luteolin in a mouse model [105].

Silymarin reduced anti-TB drug-induced hepatotoxicity in randomized clinical trials [98,99]. Lee YY in a recently published case report has also shown hepatoprotective effects of silymarin in a patient with TB [132]. Restoration of the superoxide dismutase pool is one possible mechanism for the hepatoprotective effects [99]. However, some other clinical trials and a population-based cohort analysis did not show any hepatoprotective effect of silymarin [133,134,135,136,137].

Berberine, commonly used for diabetes, high cholesterol levels in blood, and high blood pressure in ayurvedic medicine protects against isoniazid-induced liver injury in rats by reducing oxidative stress and inflammation through the upregulation of peroxisome proliferator-activated receptor γ and subsequent suppression of NF-κβ, Inducible Nitric Oxide Synthase (iNOS), and secretion of proinflammatory cytokines TNF-α and IL-1β [110].

#### 3.2.2. Potential Side Effects and Challenges Associated with Plant-Derived Natural Products

Although phytochemicals hold a huge potential as HDT candidates for TB (summarized in Table 2), the side effects of these products and challenges related to chemical stability, solubility, and bioavailability need to be addressed before their clinical application.

##### Adverse Effects

EGCG has been widely promoted as food supplement product, raising concern on its safety. The most notable adverse effect is hepatotoxicity, and the effect was dose-dependent [138,139,140]. Further, a high dose of EGCG may cause nephrotoxicity, affect ocular tissues, corneas, retinas, destroy islet β-cells, and decrease insulin sensitivity, resulting in type 1 diabetes [140]. However, in some randomized clinical trials (RCTs), EGCG was found to be safe and well tolerated even at high doses and long treatment durations [141,142,143,144]. Oral intake of quercetin in humans appears to be safe and well tolerated [145,146,147,148]. In fact, high-purity quercetin is regarded as “Generally Recognized As Safe” (GRAS) by the American Food and Drug Administration (FDA) under the intended conditions of use. However, based on the results obtained from animal studies and considering the uncertainties due to the limited human data, certain potential risk groups have been identified, e.g., patients with kidney dysfunction [145]. Reports on adverse events of silymarin are scarce. In a recent systematic review and meta-analysis, the frequency of side effects of silymarin was similar and uncommon in the intervention and non-intervention groups of patients with non-alcoholic fatty liver disease [149]. In a phase 1 and pharmacokinetic study in prostate cancer patients, silymarin was found to be well tolerated, with the most prominent adverse event being hyperbilirubinemia in 9 out of 13 patients and grade 3 elevation of alanine aminotransferase (ALT) in one patient [150]. Moreover, silymarin did not cause any notable adverse events compared to those in a placebo treatment group in hospitalized patients with COVID-19 [151]. With a long-established safety record, a good safety profile of curcumin has been found in animals and humans, even at doses up to 8 g/day; the FDA has declared this substance as GRAS [152]. In several RCTs in patients with different ailments, oral intake of curcumin did not produce any toxic effects [153,154,155,156,157,158]. Even a daily dose of 12g was safe in other phase 1 trials [152]. Only a few incidences of digestive disorders, dermatitis and allergies, lung cancer, and reduction of sperm fertility have been reported. As shown in many RCTs, resveratrol, is generally well tolerated [159,160,161,162]. However, some adverse effects, including nephrotoxicity and gastrointestinal problems, were reported in human subjects [161,163]. Long-term intake of resveratrol, which acts as a goitrogen, may lead to thyroid disruption. In overweight older adults, a higher dose of resveratrol was shown to raise the biomarkers of cardiovascular disease (CVD) risk [164]. The use of berberine in healthy volunteers or patients with different diseases was found to be safe in some clinical trials [165,166,167,168,169]; however, some other clinical trials and toxicological studies in animals have shown that berberine is toxic to gastrointestinal function, immune function, and the heart [170,171,172,173]. There is a lack of information on the toxicity profile of luteolin, gingerol, and piperine in humans. Luteolin appears to have a very good safety profile, based on the available preclinical and clinical data [174,175,176,177]. Limited clinical trials have demonstrated a good safety profile of gingerol except for mild gastrointestinal problems and heartburn [178,179,180]. Piperine is generally considered beneficial to human health except for some minor side effects, including loss of potassium, acid reflux, constipation, and nausea. However, in animal models, effects on liver, kidney, lung, embryo, and sperm quality have been observed [181,182]. To our knowledge, no studies have so far evaluated the toxicity of bergenin and allicin in humans; studies in animals and in vitro studies have demonstrated that bergenin and its natural derivatives had no significant cytotoxicity effects [183]. Severe cell damage in isolated rat liver is inflicted by allicin [184]. Allicin may lead to adverse reactions such as intolerance, allergy, gastrointestinal disturbances, and those affecting the autonomic nervous system in experimental animals [185].

Another concern regarding adverse effects for some of the phytochemicals are drug–drug interactions. The bioavailability of different drugs can be increased (e.g., paracetamol, calcium channel blockers, and antihypertensive drugs) or decreased (e.g., cholesterol-lowering drugs and immunosuppressive drugs) by quercetin [145]. By increasing drug bioavailability, quercetin may cause an upsurge in the effectiveness of a drug, but may also raise the possibility of adverse drug effects. On the other hand, by reducing drug bioavailability, quercetin would reduce the effectiveness of the drugs. Curcumin can enhance the effect of antiplatelet drugs, which may lead to hemorrhages. On the other hand, it can reduce the effectiveness of antacids [152]. By interacting with other drugs, resveratrol may attenuate the activities of these drugs or overexpression of drug transporters and CYP450 enzymes, the major cellular system involved in drug metabolism [163]. Piperine can interact with other drugs and in most cases can increase the bioavailability and consequent bioactivity of these drugs (e.g., antihypertensive, bronchodilatory, muscle relaxant, and sedative drugs); however, such an increase in activity can be harmful depending on the drug and the degree of interaction [182]. The bioavailability of some other drugs (e.g., anticoagulants and calcium channel blockers) can be reduced by piperine [182].

##### Challenges Regarding Chemical Stability and Bioavailability

A major challenge for the clinical application of the natural products including phytochemicals is their chemical instability and poor systemic bioavailability. EGCG is highly degradable under physiologic environments and displays poor systemic bioavailability, a lower absorption rate, and low membrane permeability [186]. Since EGCG has a low half-life in plasma and the outflow rate is much higher compared to the influx, the production of an efficient therapeutic potential in the target site require a high dose of EGCG, which can be toxic. Quercetin has a poor bioavailability, less than 10% of what is consumed, due to its low water solubility, chemical stability, and absorption in the gastrointestinal system, interactions with other compounds, e.g., fats and pectin, and the influence of gut microbiota [187,188]. Low bioavailability, rapid metabolism, weak absorption due to low water solubility, and instability in higher pH and temperature are the most limiting factors for the use of resveratrol as a pharmaceutical drug [161]. Luteolin is also well known for its low oral bioavailability due to its low water solubility and rapid and extensive metabolism [175]. However, metabolites of luteolin are also biologically active, possessing anti-inflammatory activity. Moreover, the recycling of luteolin can contribute to the bioactivity of luteolin. Biological activities of metabolites of other polyphenols, including flavonoids, and the enterohepatic recycling of flavonoids are also evident [175]. The clinical applications of silymarin, curcumin, bergenin, gingerol, berberine, and piperine are also limited by their low water solubility, poor oral bioavailability, chemical instability, and low absorption in the gastrointestinal tract [171,189,190,191,192,193]. Low water solubility, instability, and poor bioavailability also greatly limit the bioefficacies of allicin. Although allicin is rapidly metabolized, it was still bioavailable in in vitro experimental models as the lipid soluble property of this organosulfur compound allows it to rapidly cross cell membranes [185].

To overcome the limitations of poor water solubility and bioavailability, several novel approaches have been introduced that include (i) prodrug approaches i.e., synthesis of chemically modified agents that transform to release active drugs, retaining their bio-efficacy, (ii) improved formulations and delivery systems, including nano-encapsulations in liposomes, micelles and exosome-like nanoparticles, microneedle-mediated intradermal delivery, micro/nano emulsions, incorporation in lipid-based carriers, complexation with other molecules such as cyclodextrin and phospholipids, and cocrystallization for efficient penetration and better bioavailability and biodistribution [102,161,171,175,181,185,186,187,188,189,191,192,193,194,195]. Prodrug and nanoparticle formulation approaches have been tested for the treatment of tuberculosis by curcumin. Monocarbonyl analogs of curcumin were synthesized and several of these analogs remarkably reduced the number *M. tuberculosis* and *M. marinum* (Mm) [194]. Nanoparticle-formulated curcumin exhibited a five-fold increase in the bioavailability of curcumin in mice [102] and effectively reversed the antitubercular drug-mediated hepatotoxicity, augmented the clearance of *M. tuberculosis* from infected macrophages, lowered the incidence of reinfection and reactivation, and improved the host protective ability of BCG [102,119,122]. These sophisticated techniques can be tailored to suit specific routes of administration and tissue targets, and need to be tested in large-scale clinical trials, including trials in tuberculosis.

## 4. Comparison of Therapeutic Potential of HDT Candidates

Many licensed medicines and nutrients have long been used as adjunct treatments of complex diseases such as TB. However, the concept of host-directed therapy itself is relatively new; studies on the mechanism of action of adjunct therapies has led to the generation of this concept. Anti-inflammatory drugs and lipid-lowering statins used as HDT for TB treatment seem to be at an advanced stage for application in mainstream therapy. Aspirin, or acetylsalicylic acid, a non-steroidal anti-inflammatory drug (NSAID), alone or in combination with other drugs (ibuprofen or corticosteroid) has been tested in phase 2 trials. Of the completed trials, the results showed promising findings in terms of reduced inflammation; some studies also showed a favorable clinical outcome particularly in TB meningitis [45,48,49]. Dexamethasone, a corticosteroid, alone did not exhibit any beneficial clinical outcome, but together with aspirin showed some benefits in the reduction of inflammation [48]. Sulfasalazine, belonging to the aminosalicylates group (NSAID), in HDT showed favorable outcomes without any deaths or treatment failures in XFR-TB [52] and deserves further evaluation in phase 4 trials. The antibiotic doxycycline, when used as an adjunct therapy, has shown significant improvement in lung function and reduced pulmonary cavity volume in pulmonary TB and was safe [54,55]. The positive findings limiting TB immunopathology warrant larger phase 3 trials. Lipid-lowering statins (atorvastatins and pravastatin) and metformin (to treat type 2 diabetes) have been used in a number of phase 2 trials. The studies showed reductions in inflammatory markers, limiting lung tissue damage, and the induction of autophagy and apoptosis of infected cells, but these drugs did not speed up sputum culture conversion. The majority of the HDT trials have been phase 2 studies, and only four of these have entered phase 3 trials (vitamin D, aspirin, dexamethasone, and doxycycline). It remains to be seen whether these trials will show clinically favorable effects on the treatment of TB or be applicable in the mainstream clinical management of TB.

The phytochemicals described in this review are promising HDT candidates for TB. However, clinical research on use of phytochemicals as anti-TB drugs is still in its infancy; only a few candidates, e.g EGCG, piperine, quercetin, and silymarin, have reached the clinical trial stage. Piperine, being used in a phase 3 trial, has reached to the most advanced stage as an anti-TB HDT. Moreover, apart from the HDT activities, piperine can directly act on the Mtb inside the host cells. Since its interaction with other drugs increases the bioavailability and bioactivity of these drugs, it has the potential to be used in synergy with other drugs and/or phytochemicals [196]. However, although generally regarded as safe, the safety profile of piperine has to be scientifically proved in large-scale clinical trials. Quercetin, with its pleiotropic HDT activities, antimycobacterial activities, and very good safety profile as recognized by the FDA, could be a drug of choice for the treatment of tuberculosis and has already entered into a phase 2 clinical trial. The multitargeted activities of EGCG along with its antimycobacterial activities may also make it a potential therapeutic candidate, and it has also undergone a randomized clinical trial. However, the toxic effects of EGCG are a major concern for its therapeutic use in TB, and the safe and effective dose needs to optimized in further clinical trials. Another phytochemical that has been tested as an anti-TB drug in a randomized clinical trial is silymarin, but its toxicity profile is limited. To our knowledge, no clinical trial of curcumin as an anti-TB therapy has been performed so far; however, it has a well-established safety profile in both animals and humans. Moreover, as discussed in the previous section, analogs of curcumin (prodrug approach) and nanoparticle-formulated curcumin have been successfully used in the treatment of TB, putting it one step further forward than other phytochemicals.

## 5. Concluding Remarks and Future Perspectives

Host-directed therapy possesses revolutionary potential for treating TB, considering the emergence of alarmingly high rates of AMR strains of Mtb and the limited repertory of antibiotics. HDT, by regulating different host functions including phagocytosis, autophagy, apoptosis (death pathways), the modulation of immune and inflammatory responses, and metabolic pathways, can be effective against MDR/XDR Mtb strains. Additionally, it is effective against metabolically inactive and non-replicating bacilli inside granuloma during latent tuberculosis infections, which are tolerant or insensitive to current standard antibiotics. HDTs are less likely to result in drug resistance as there is no direct selection pressure on mycobacteria. Moreover, the use of HDTs as adjuncts to conventional antibiotics has been suggested to exert synergistic or additive effects, resulting in a reduction of treatment time and/or lowering of antibiotic doses, thereby reducing drug toxicity. In addition, HDT may reinstitute the balance between immune and inflammatory responses, which has been disrupted by Mtb infection. HDT thus may reduce disease pathology and improve overall treatment outcomes for TB patients.

The use of repurposed drugs as HDTs, with proven safety profiles in humans, although in other clinical conditions, have the potential to be quickly implemented to respond to the ever-increasing threat of antibiotic resistance in Mtb. This strategy will also reduce the huge investments of money, time, and labor in the development of new drugs. Many of the repurposed drugs have already entered into different phases of anti-TB clinical trials (Table 1). On the other hand, natural products containing many bioactive compounds are excellent sources for generating novel and effective anti-TB drugs due to their remarkable chemical and structural diversity. Plant-derived natural products or phytochemicals in the established settings of traditional ayurvedic treatments provide attractive and efficient alternatives or adjunct therapies to conventional anti-TB treatments. They offer potential for not only eliminating bacteria but also alleviating the side effects of standard anti-TB drugs by reducing the immunopathology of lungs and hepatotoxicity.

Although HDT offers a ray of hope and encouragement for expanded treatment possibilities, there remain significant challenges that need to be addressed before the clinical application of HDT candidates in TB. These are safety and toxicity, drug–drug interactions, variations in effectiveness, and validating clinical efficiency. Many of the HDT candidates are shown to be effective in vitro or preclinical studies, but evidence from human studies or clinical trials is limited and often conflicting. Factors causing distinct results include but are not limited to study design, sample size, sociodemographic and behavioral factors, food habits, malnutrition, dose selection, route of administration, geographical location, ethnicity, coinfection, immune suppression, metabolic disorders, and genetic pre-disposition associated with susceptibility/resistance to infection. New and advanced phases of clinical trials, preferably multicenter with harmonized and meticulous design, enrolling different populations of clinical relevance, and applying homogeneous protocols through existent TB networks are needed for the validation of the clinical efficiency of HDT candidates. Given the recent advancements in research and development, HDT candidates including re-purposed drugs and plant products applied as adjuncts to standard antibiotic therapy will present a crucial breakthrough in the treatment of TB.

## Figures and Tables

**Table 1:** Clinical trials of repurposed drugs for host-directed therapy in the treatment of tuberculosis.

FDA Approved Drugs	Clinical Indications	Probable Mechanisms and Outcomes/Endpoints	Study Site, Trial Phase, Sample Size	References
Targeting cell death				
Phenylbutyrate, Vitamin D3	Urea cycle disorders, healthy bones, muscles, nerves, and to support the immune system	Induces LL-37 (antimicrobial peptide) expression, autophagy, reduction of proinflammatory cytokines/chemokines, inhibition of ER-stress related genes, intracellular killing of Mtb, and early sputum culture negative	Bangladesh Phase 2 *n* = 288	A Mily, 2015 [39] NCT01580007 RS Rekha, 2018 [40]
Phenylbutyrate, Vitamin D3	As above	Mitigate clinical TB symptoms and disease-specific complications	Ethiopia Phase 2 n = 348	Bekele, 2018 [41] NCT01698476
Vitamin D3	Immunomodulatory, anti-inflammatory, modulation of cell growth, neuromuscular function, glucose metabolism	Faster resolution of fever, cough, and improvement in nutritional status	Indonesia Phase 2 *n* = 84	L Tamara 2022 [42] NCT05073965
Imatinib	Chronic myelogenous leukemia and other cancers	Human trial will assess safety, optimum dose identification, maximize bactericidal activity by immune cells; induce myelopoiesis; time to sputum culture conversion	Nepal and Vietnam Phase 2 *n* = 72 *n* = 180	CR Giver, 2019 [43] Cleverley TL, 2023 [44] IMPACT-TB* NCT03891901
Vitamin D3	As above	Spondylitis Tuberculosis; clinical outcomes, serum levels of TLR-2, and TLR-4	Indonesia Phase 2 Phase 3 n = 37	Jainal Arifin, Firdaus Hamid, Andi Alfian Zainuddin NCT05376189 Not yet recruiting
Anti-inflammatory				
Acetylsalicylic acid (Aspirin)	Pain, inflammation or arthritis, risk of heart attack, stroke or blood clot	Favorable outcome in high dose group	South Africa Phase 2 n = 146	JF Schoeman, 2011 [45]
Aspirin	As above	Primary outcome is all-cause death between inclusion and week 40	Ivory Coast, Madagascar, Uganda, and South Africa Phase 3 n = 768	Thomas Maitre, 2022 [46] NCT04145258 INTENSE-TBM
Aspirin	As above	To shorten the duration and improve the treatment outcomes; apply anti-inflammatory approaches to improve disability-free survival	India Phase 2 n = 372	LR Inbaraj, 2024 [47] NCT05917340
Aspirin, Dexamethasone	Pain, inflammation or arthritis, risk of heart attack, stroke or blood clot	Significant inhibition of pro-thrombotic TXA2 and proinflammatory prostaglandins (PGD2, PGE2, and PGF2) and upregulation of pro-resolution protectins in TB meningitis	Vietnam Phase 2 n = 120	NTH Mai, 2018 [48]
Aspirin Corticosteroid	Anti-inflammatory, immune-modulatory, rheumatoid arthritis, lupus or vasculitis	Anti-aggregation, anti-inflammatory, and antioxidant properties, and an antithrombotic effect	India Phase 2 n = 153	UK Misra, 2018 [49]
Aspirin, Ibuprofen	Reducing pain, fever, inflammation, rheumatoid disorders, dysmenorrhea, and osteoarthritis	Inhibitors of cyclooxygenase-1 and cyclooxygenase-2, which are involved in synthesis of prostaglandins and leukotrienes	South Africa and Georgia Phase 2b n = 354	L Arias, 2023 [50] NCT04575519
Ibuprofen	-	Potential efficacy and safety when used as adjunctive therapy in XDR-TB patients; immune responses	Georgia, South Africa Phase 2 n = 24	Cris Vilaplana NCT02781909
Dexamethasone	Inflammation, certain forms of arthritis; severe allergies; asthma; certain types of cancer	Immune reconstitution inflammatory syndrome (IRIS); no favorable outcome	Vietnam Indonesia Phase 2 n = 520	J Donova, 2023 [51] NCT03092817
Dexamethasone	As above	In TB meningitis. Outcomes: Survival rate; Incidence of new neurological events; disability, frequency of severe and serious adverse events; need for rescue corticosteroids.	Vietnam Phase 3 n = 720	Guy Thwaites NCT03100786
Sulfasalazine	Lower inflammation in certain diseases and help prevent the need for steroids; used in ulcerative colitis, rheumatoid arthritis	Effective, safe, well-tolerated, and cost-effective; no treatment failure or death	China Phase 2 n = 44	Liang Fu, 2024 [52] ChiCTR2000032298
Immune-modulating				
Etoricoxib	Cyclooxygenase-2 inhibitor	Reduced circulating vaccine-responsive T-cells	Norway Phase 1/2 n = 222	S Jenum, 2021 [53] NCT02503839
Doxycycline	Preventing the growth and spread of bacteria, MMP inhibitor	MMP-1, -8, -9, -12 and -13, suppressed type I collagen, elastin destruction, reduced pulmonary cavity volume	Singapore Phase 2 n = 30	QH Miow 2021 [54] NCT02774993
Doxycycline	As above	Outcome: improvement of lung function and decrease in tissue destruction	Singapore, Malaysia Phase 3 n = 150	J Mi, 2024 [55] NCT05473520
N-acetylcysteine (NAC)	Treat acetaminophen overdose	Restore reduced form of glutathione, exert antioxidant effects, decreased peroxidation	Brazil Phase 2 n = 39	IP Safe, 2021 [56] NCT03281226 RIPENACTB
N-acetylcysteine (NAC)	As above	Increased glutathione levels; improved recovery of lung function; no impact on sputum culture conversion	Tanzania Phase 2 n = 140	RS Wallis, 2024 [57] NCT03702738 TB SEQUEL
Regulating metabolic pathways				
Statins Atorvastatin	Hypercholesterolemia	Induces phagosome and phagolysosome maturation, autophagy and apoptosis in Mtb-infected PBMCs	Cape Town Phase 2b n = 220	K Wolmarans, 2022 NCT04147286 Poster
Rosuvastatin	Hypercholesterolemia, to prevent cardiovascular disease	Safe but did not demonstrate substantial benefits on culture conversion	Philippines, Vietnam, Uganda Phase 2b n = 137	GB Cross, 2023 [58] NCT04504851
Pravastatin	Hypercholesterolemia	Inhibits cellular cholesterol biosynthesis, phagolysosome maturation and induces autophagy	South Africa Phase 2 n = 16	SP Parihar, 2019 [59] NCT03882177 StAT-TB
Pravastatin	Hypercholesterolemia	Safety and tolerance, severity of adverse outcomes	South Africa Phase 2b n = 40	PC Karakousis; RE Chaisson; N Martinson, NCT03456102 StAT-TB
Atorvastatin	Hypercholesterolemia	Dose finding, outcome: Speedier sputum culture conversion, adverse events	Nigeria Phase 2 n = 440	Olanisun O Adewole NCT06199921 StatinTB
Metformin	Diabetes	Did not speed up sputum culture conversion, reduced inflammatory markers and lung tissue damage. Reduced inflammatory chemokines.	India Phase 2 n = 322	C Padmapriydarsini 2022 [60] CTRI/2018/01/011176 NP Kumar 2024 [61], H Krismawati 2024 [62]
Metformin	As above	Outcomes: % participants experiencing leprosy reactions; frequency of serious adverse events	Indonesia Phase 2 n = 166	H Krismawati 2024 NCT05243654 MetLep Trial
Metformin	As above	Outcomes: Safety and tolerability; efficacy as measured by time to sputum conversion	South Africa Phase 2 n = 112	Hardy Kornfeld NCT04930744 Recruiting
Everolimus, auranofin, CC-11050, vitamin D2	Renal cell carcinoma	CC-11050 and everolimus arms were safe and relatively well tolerated; had increased recovery of FEV1 at day 180	South Africa Phase 2 n = 200	RS Wallis, 2021 [63] NCT02968927
Everolimus	Renal cell carcinoma	Modulate autophagy via the inhibition of mTOR; higher recovery of lung function at day 180; peak glycolytic activity was reduced	South Africa Phase 2 Secondary analysis	RS Wallis, 2022 [64]

IMPACT-TB*, Imatinib Mesylate per Oral As a Clinical Therapeutic for TB.

**Table 2:** Phytochemicals in clinical or preclinical trials as potential host-directed therapeutic candidates for the treatment of tuberculosis.

Phytochemicals	Categories (Source)	HDT Effects for TB	Developmental Stage as HDT for TB	References
Epigallocatechin gallate (EGCG)	Polyphenol (Green tea)	Induction of autophagy, phagosome maturation through autophagic flux, and reduction of inflammation and oxidative stress	Randomized clinical trial	Agarwal A, 2010 [96]
Quercetin	Polyphenolic flavonoid (Capers, red onions, kale)	Reduction of caseous necrosis of hepatocytes, inhibition of inflammatory mediators, suppression of oxidative stress, and boosting of endogenous antioxidants	Phase 2 clinical trial	Butov D, 2016 [97]
Silymarin	Polyphenolic flavonoid (Milk thistle seeds)	Reduction of anti-TB drug-induced hepatotoxicity and induction of Th-1 cytokines	Randomized double-blind clinical trial	Talebi A, 2023 [98], Luangchosiri C, 2015 [99]
Curcumin	Polyphenol (Turmeric)	Induction of apoptosis, autophagy, phagosome lysosome fusion, attenuation of isoniazide-induced hepatotoxicity, downregulation of oxidative stress and inflammation, reversion of isoniazide-induced dysfunction and apoptosis of T cells, restoration of isoniazide-induced suppression of antigen-specific cytokine responses, activation of Th and Tc cells, enhanced generation of central memory T cells, and reduction of the release of proinflammatory cytokines IL-6 and TNF-α	Preclinical trial (mouse model)	Lara-Espinosa JV, 2022 [100], Gupta PK, 2023 [101] Tousif S, 2017 [102], Li Y, 2022 [103]
Resveratrol	Stilbenoid polyphenol (Peanuts, grapes, cranberries)	Inhibition of Mtb-induced apoptosis and reversion of Mtb-induced secretion of IL-6 and TNF-α	Preclinical (mouse model)	Yang H, 2019 [104]
Luteolin	Polyphenolic flavonoid (Herbs, fruits and vegetables)	Alleviation of isoniazide-induced hepatotoxicity, promotion of central memory T cells, activation of NK and NKT cells, and induction of Th1 (IFN-γ and TNF-α) and Th17 (IL-17 and IL-22) cytokines	Preclinical (mouse model)	Singh DK, 2021 [105]
Bergenin	Polyphenol (Different parts of many plants)	Stimulation of Th1 and Th17 response and promotion of antigen-specific central memory T cell	Preclinical (mouse model)	Dwivedi VP, 2017 [106] Kumar S, 2019 [107]
6-Gingerol	Polyphenol (Ginger)	Promotion of Th1/Th17 responses	Preclinical (mouse model)	Bhaskar A, 2020 [108]
Piperine	Alkaloid (Black pepper)	Promotion of the proliferation of T and B cells, Th-1 cytokines, and macrophage activation	Phase 3 clinical trial	Patel N, 2017 [109]
Berberine	Isoquinoline alkaloid (Medicinal plants)	Protection from isoniazide -induced liver injury, reduction of oxidative stress and inflammation, induction of macrophage activation, and Th1/Th17 polarization, T effector memory, T central memory, and tissue-resident memory T cell responses	Preclinical (mouse and rat models)	Mahmoud AM, 2014 [110], Pahuja I, 2023 [111], Ozturk M, 2021 [112]
Allicin	Organosulfur compound (Garlic)	Induction of protective Th1 response and reduction of Mtb-induced ROS and TNF-α	Preclinical (mouse model)	Dwivedi VP, 2019 [113]

## References

[1] (2024). WHO Guidelines Approved by the Guidelines Review Committee. WHO Consolidated Guidelines on Tuberculosis: Module 6: Tuberculosis and Comorbidities.

[2] Singh R., Dwivedi S.P., Gaharwar U.S., Meena R., Rajamani P., Prasad T. (2020). Recent updates on drug resistance in *Mycobacterium tuberculosis*. J. Appl. Microbiol..

[3] Bodke H., Wagh V., Kakar G. (2023). Diabetes Mellitus and Prevalence of Other Comorbid Conditions: A Systematic Review. Cureus.

[4] Nyasulu P.S., Doumbia C.O., Ngah V., Togo A.C.G., Diarra B., Chongwe G. (2024). Multidrug-resistant tuberculosis: Latest opinions on epidemiology, rapid diagnosis and management. Curr. Opin. Pulm. Med..

[5] Diriba G., Alemu A., Yenew B., Tola H.H., Gamtesa D.F., Mollalign H., Eshetu K., Moga S., Abdella S., Tollera G. (2023). Epidemiology of extensively drug-resistant tuberculosis among patients with multidrug-resistant tuberculosis: A systematic review and meta-analysis. Int. J. Infect. Dis..

[6] WHO (2023). Global Tuberculosis Report.

[7] Ahmed S., Raqib R., Guethmundsson G.H., Bergman P., Agerberth B., Rekha R.S. (2020). Host-Directed Therapy as a Novel Treatment Strategy to Overcome Tuberculosis: Targeting Immune Modulation. Antibiotics.

[8] Bergman P., Raqib R., Rekha R.S., Agerberth B., Gudmundsson G.H. (2020). Host Directed Therapy Against Infection by Boosting Innate Immunity. Front. Immunol..

[9] Kaufmann S.H.E., Dorhoi A., Hotchkiss R.S., Bartenschlager R. (2018). Host-directed therapies for bacterial and viral infections. Nat. Rev. Drug Discov..

[10] Bussi C., Gutierrez M.G. (2019). *Mycobacterium tuberculosis* infection of host cells in space and time. FEMS Microbiol. Rev..

[11] Ravesloot-Chavez M.M., Van Dis E., Stanley S.A. (2021). The Innate Immune Response to *Mycobacterium tuberculosis* Infection. Annu. Rev. Immunol..

[12] Rodriguez-Carlos A., Jacobo-Delgado Y., Santos-Mena A.O., Garcia-Hernandez M.H., De Jesus-Gonzalez L.A., Lara-Ramirez E.E., Rivas-Santiago B. (2023). Histone deacetylase (HDAC) inhibitors- based drugs are effective to control *Mycobacterium tuberculosis* infection and promote the sensibility for rifampicin in MDR strain. Mem. Inst. Oswaldo Cruz.

[13] Mihret A. (2012). The role of dendritic cells in *Mycobacterium tuberculosis* infection. Virulence.

[14] Prendergast K.A., Kirman J.R. (2013). Dendritic cell subsets in mycobacterial infection: Control of bacterial growth and T cell responses. Tuberculosis.

[15] Silva Miranda M., Breiman A., Allain S., Deknuydt F., Altare F. (2012). The tuberculous granuloma: An unsuccessful host defence mechanism providing a safety shelter for the bacteria?. Clin. Dev. Immunol..

[16] Yang C.T., Cambier C.J., Davis J.M., Hall C.J., Crosier P.S., Ramakrishnan L. (2012). Neutrophils exert protection in the early tuberculous granuloma by oxidative killing of mycobacteria phagocytosed from infected macrophages. Cell Host Microbe.

[17] Jena P., Mohanty S., Mohanty T., Kallert S., Morgelin M., Lindstrom T., Borregaard N., Stenger S., Sonawane A., Sorensen O.E. (2012). Azurophil granule proteins constitute the major mycobactericidal proteins in human neutrophils and enhance the killing of mycobacteria in macrophages. PLoS ONE.

[18] Dallenga T., Repnik U., Corleis B., Eich J., Reimer R., Griffiths G.W., Schaible U.E.M. (2017). tuberculosis-Induced Necrosis of Infected Neutrophils Promotes Bacterial Growth Following Phagocytosis by Macrophages. Cell Host Microbe.

[19] Lu C.C., Wu T.S., Hsu Y.J., Chang C.J., Lin C.S., Chia J.H., Wu T.L., Huang T.T., Martel J., Ojcius D.M. (2014). NK cells kill mycobacteria directly by releasing perforin and granulysin. J. Leukoc. Biol..

[20] Larsen S.E., Williams B.D., Rais M., Coler R.N., Baldwin S.L. (2022). It Takes a Village: The Multifaceted Immune Response to *Mycobacterium tuberculosis* Infection and Vaccine-Induced Immunity. Front. Immunol..

[21] Lewinsohn D.M., Lewinsohn D.A. (2022). The Missing Link in Correlates of Protective Tuberculosis Immunity: Recognizing the Infected Cell. Front. Immunol..

[22] Lin P.L., Flynn J.L. (2015). CD8 T cells and *Mycobacterium tuberculosis* infection. Semin. Immunopathol..

[23] Aerts L., Selis E., Corbiere V., Smits K., Van Praet A., Dauby N., Petit E., Singh M., Locht C., Dirix V. (2019). HBHA-Induced Polycytotoxic CD4(+) T Lymphocytes Are Associated with the Control of *Mycobacterium tuberculosis* Infection in Humans. J. Immunol..

[24] Qin S., Chen R., Jiang Y., Zhu H., Chen L., Chen Y., Shen M., Lin X. (2021). Multifunctional T cell response in active pulmonary tuberculosis patients. Int. Immunopharmacol..

[25] Maglione P.J., Xu J., Chan J. (2007). B cells moderate inflammatory progression and enhance bacterial containment upon pulmonary challenge with *Mycobacterium tuberculosis*. J. Immunol..

[26] Augenstreich J., Arbues A., Simeone R., Haanappel E., Wegener A., Sayes F., Le Chevalier F., Chalut C., Malaga W., Guilhot C. (2017). ESX-1 and phthiocerol dimycocerosates of *Mycobacterium tuberculosis* act in concert to cause phagosomal rupture and host cell apoptosis. Cell Microbiol..

[27] Manzanillo P.S., Ayres J.S., Watson R.O., Collins A.C., Souza G., Rae C.S., Schneider D.S., Nakamura K., Shiloh M.U., Cox J.S. (2013). The ubiquitin ligase parkin mediates resistance to intracellular pathogens. Nature.

[28] Watson R.O., Manzanillo P.S., Cox J.S., Extracellular M. (2012). tuberculosis DNA targets bacteria for autophagy by activating the host DNA-sensing pathway. Cell.

[29] Braian C., Hogea V., Stendahl O. (2013). *Mycobacterium tuberculosis*- induced neutrophil extracellular traps activate human macrophages. J. Innate Immun..

[30] Jeong E.K., Lee H.J., Jung Y.J. (2022). Host-Directed Therapies for Tuberculosis. Pathogens.

[31] Brandenburg J., Marwitz S., Tazoll S.C., Waldow F., Kalsdorf B., Vierbuchen T., Scholzen T., Gross A., Goldenbaum S., Holscher A. (2021). WNT6/ACC2-induced storage of triacylglycerols in macrophages is exploited by *Mycobacterium tuberculosis*. J. Clin. Investig..

[32] Wik J.A., Skalhegg B.S. (2022). T Cell Metabolism in Infection. Front. Immunol..

[33] Tiberi S., du Plessis N., Walzl G., Vjecha M.J., Rao M., Ntoumi F., Mfinanga S., Kapata N., Mwaba P., McHugh T.D. (2018). Tuberculosis: Progress and advances in development of new drugs, treatment regimens, and host-directed therapies. Lancet Infect. Dis..

[34] Huang X., Lowrie D.B., Fan X.Y., Hu Z. (2024). Natural products in anti-tuberculosis host-directed therapy. Biomed. Pharmacother..

[35] Wallis R.S., O’Garra A., Sher A., Wack A. (2023). Host-directed immunotherapy of viral and bacterial infections: Past, present and future. Nat. Rev. Immunol..

[36] Hawn T.R., Shah J.A., Kalman D. (2015). New tricks for old dogs: Countering antibiotic resistance in tuberculosis with host-directed therapeutics. Immunol. Rev..

[37] Guler R., Brombacher F. (2015). Host-directed drug therapy for tuberculosis. Nat. Chem. Biol..

[38] Cubillos-Angulo J.M., Nogueira B.M.F., Arriaga M.B., Barreto-Duarte B., Araujo-Pereira M., Fernandes C.D., Vinhaes C.L., Villalva-Serra K., Nunes V.M., Miguez-Pinto J.P. (2022). Host-directed therapies in pulmonary tuberculosis: Updates on anti-inflammatory drugs. Front. Med..

[39] Mily A., Rekha R.S., Kamal S.M., Arifuzzaman A.S., Rahim Z., Khan L., Haq M.A., Zaman K., Bergman P., Brighenti S. (2015). Significant Effects of Oral Phenylbutyrate and Vitamin D3 Adjunctive Therapy in Pulmonary Tuberculosis: A Randomized Controlled Trial. PLoS ONE.

[40] Rekha R.S., Mily A., Sultana T., Haq A., Ahmed S., Mostafa Kamal S.M., van Schadewijk A., Hiemstra P.S., Gudmundsson G.H., Agerberth B. (2018). Immune responses in the treatment of drug-sensitive pulmonary tuberculosis with phenylbutyrate and vitamin D(3) as host directed therapy. BMC Infect. Dis..

[41] Bekele A., Gebreselassie N., Ashenafi S., Kassa E., Aseffa G., Amogne W., Getachew M., Aseffa A., Worku A., Raqib R. (2018). Daily adjunctive therapy with vitamin D(3) and phenylbutyrate supports clinical recovery from pulmonary tuberculosis: A randomized controlled trial in Ethiopia. J. Intern. Med..

[42] Tamara L., Kartasasmita C.B., Alam A., Gurnida D.A. (2022). Effects of Vitamin D supplementation on resolution of fever and cough in children with pulmonary tuberculosis: A randomized double-blind controlled trial in Indonesia. J. Glob. Health.

[43] Giver C.R., Shaw P.A., Fletcher H., Kaushal D., Pamela G., Omoyege D., Bisson G., Gumbo T., Wallis R., Waller E.K. (2019). IMPACT-TB*: A Phase II Trial Assessing the Capacity of Low Dose Imatinib to Induce Myelopoiesis and Enhance Host Anti-Microbial Immunity Against Tuberculosis. *Imatinib Mesylate per Oral As a Clinical Therapeutic for TB. Blood.

[44] Cleverley T.L., Peddineni S., Guarner J., Cingolani F., Garcia P.K., Koehler H., Mocarski E.S., Kalman D. (2023). The host-directed therapeutic imatinib mesylate accelerates immune responses to Mycobacterium marinum infection and limits pathology associated with granulomas. PLoS Pathog..

[45] Schoeman J.F., Janse van Rensburg A., Laubscher J.A., Springer P. (2011). The role of aspirin in childhood tuberculous meningitis. J. Child. Neurol..

[46] Maitre T., Bonnet M., Calmy A., Raberahona M., Rakotoarivelo R.A., Rakotosamimanana N., Ambrosioni J., Miro J.M., Debeaudrap P., Muzoora C. (2022). Intensified tuberculosis treatment to reduce the mortality of HIV-infected and uninfected patients with tuberculosis meningitis (INTENSE-TBM): Study protocol for a phase III randomized controlled trial. Trials.

[47] Inbaraj L.R., Manesh A., Ponnuraja C., Bhaskar A., Srinivasalu V.A., Daniel B.D. (2024). Comparative evaluation of intensified short course regimen and standard regimen for adults TB meningitis: A protocol for an open label, multi-center, parallel arms, randomized controlled superiority trial (INSHORT trial). Trials.

[48] Mai N.T.H., Dobbs N., Phu N.H., Colas R.A., Thao L.T.P., Thuong N.T.T., Nghia H.D.T., Hanh N.H.H., Hang N.T., Heemskerk A.D. (2018). A randomised double blind placebo controlled phase 2 trial of adjunctive aspirin for tuberculous meningitis in HIV-uninfected adults. eLife.

[49] Misra U.K., Kalita J., Sagar B., Bhoi S.K. (2018). Does adjunctive corticosteroid and aspirin therapy improve the outcome of tuberculous meningitis?. Neurol. India.

[50] Arias L., Otwombe K., Waja Z., Tukvadze N., Korinteli T., Moloantoa T., Fonseca K.L., Pillay N., Seiphetlo T., Ouchi-Vernet D. (2023). SMA-TB: Study protocol for the phase 2b randomized double-blind, placebo-controlled trial to estimate the potential efficacy and safety of two repurposed drugs, acetylsalicylic acid and ibuprofen, for use as adjunct therapy added to, and compared with, the standard WHO recommended TB regimen. Trials.

[51] Donovan J., Bang N.D., Imran D., Nghia H.D.T., Burhan E., Huong D.T.T., Hiep N.T.T., Ngoc L.H.B., Thanh D.V., Thanh N.T. (2023). Adjunctive Dexamethasone for Tuberculous Meningitis in HIV-Positive Adults. N. Engl. J. Med..

[52] Fu L., Wang W., Xiong J., Zhang P., Li H., Zhang X., Liang H., Yang Q., Wang Z., Chen X. (2024). Evaluation of Sulfasalazine as an Adjunctive Therapy in Treating Pulmonary Pre-XDR-TB: Efficacy, Safety, and Treatment Implication. Infect. Drug Resist..

[53] Jenum S., Tonby K., Rueegg C.S., Ruhwald M., Kristiansen M.P., Bang P., Olsen I.C., Sellaeg K., Rostad K., Mustafa T. (2021). A Phase I/II randomized trial of H56:IC31 vaccination and adjunctive cyclooxygenase-2-inhibitor treatment in tuberculosis patients. Nat. Commun..

[54] Miow Q.H., Vallejo A.F., Wang Y., Hong J.M., Bai C., Teo F.S., Wang A.D., Loh H.R., Tan T.Z., Ding Y. (2021). Doxycycline host-directed therapy in human pulmonary tuberculosis. J. Clin. Investig..

[55] Mi J., Wu X., Liang J. (2024). The advances in adjuvant therapy for tuberculosis with immunoregulatory compounds. Front. Microbiol..

[56] Safe I.P., Amaral E.P., Araujo-Pereira M., Lacerda M.V.G., Printes V.S., Souza A.B., Beraldi-Magalhaes F., Monteiro W.M., Sampaio V.S., Barreto-Duarte B. (2020). Adjunct N-Acetylcysteine Treatment in Hospitalized Patients with HIV-Associated Tuberculosis Dampens the Oxidative Stress in Peripheral Blood: Results From the RIPENACTB Study Trial. Front. Immunol..

[57] Wallis R.S., Sabi I., Lalashowi J., Bakuli A., Mapamba D., Olomi W., Siyame E., Ngaraguza B., Chimbe O., Charalambous S. (2024). Adjunctive N-Acetylcysteine and Lung Function in Pulmonary Tuberculosis. NEJM Evid..

[58] Cross G.B., Sari I.P., Kityo C., Lu Q., Pokharkar Y., Moorakonda R.B., Thi H.N., Do Q., Dalay V.B., Gutierrez E. (2023). Rosuvastatin adjunctive therapy for rifampicin-susceptible pulmonary tuberculosis: A phase 2b, randomised, open-label, multicentre trial. Lancet Infect. Dis..

[59] Parihar S.P., Guler R., Brombacher F. (2019). Statins: A viable candidate for host-directed therapy against infectious diseases. Nat. Rev. Immunol..

[60] Padmapriydarsini C., Mamulwar M., Mohan A., Shanmugam P., Gomathy N.S., Mane A., Singh U.B., Pavankumar N., Kadam A., Kumar H. (2022). Randomized Trial of Metformin with Anti-Tuberculosis Drugs for Early Sputum Conversion in Adults with Pulmonary Tuberculosis. Clin. Infect. Dis..

[61] Pavan Kumar N., Padmapriyadarsini C., Nancy A., Tamizhselvan M., Mohan A., Reddy D., Ganga Devi N.P., Rathinam P., Jeyadeepa B., Shandil R.K. (2024). Effect of Metformin on systemic chemokine responses during anti-tuberculosis chemotherapy. Tuberculosis.

[62] Krismawati H., Muchtar S.V., Rahardjani M., Utami N.N., Oktaviani M., Puspatriani K., Syamsiah, Imbiri N., Hasvitasari D.E., Fajrianti D.R. (2023). Metformin as adjunctive therapy in combination with multidrug treatment for multibacillary leprosy: A protocol for a randomized double-blind, controlled Phase 2 trial in Indonesia (MetLep Trial). Wellcome Open Res..

[63] Wallis R.S., Ginindza S., Beattie T., Arjun N., Likoti M., Edward V.A., Rassool M., Ahmed K., Fielding K., Ahidjo B.A. (2021). Adjunctive host-directed therapies for pulmonary tuberculosis: A prospective, open-label, phase 2, randomised controlled trial. Lancet Respir. Med..

[64] Wallis R.S., Ginindza S., Beattie T., Arjun N., Likoti M., Sebe M., Edward V.A., Rassool M., Ahmed K., Fielding K. (2022). Lung and blood early biomarkers for host-directed tuberculosis therapies: Secondary outcome measures from a randomized controlled trial. PLoS ONE.

[65] Steiger J., Stephan A., Inkeles M.S., Realegeno S., Bruns H., Kroll P., de Castro Kroner J., Sommer A., Batinica M., Pitzler L. (2016). Imatinib Triggers Phagolysosome Acidification and Antimicrobial Activity against Mycobacterium bovis Bacille Calmette-Guerin in Glucocorticoid-Treated Human Macrophages. J. Immunol..

[66] Adikesavalu H., Gopalaswamy R., Kumar A., Ranganathan U.D., Shanmugam S. (2021). Autophagy Induction as a Host-Directed Therapeutic Strategy against *Mycobacterium tuberculosis* Infection. Medicina.

[67] Oh J.E., Lee H.K. (2014). Pattern recognition receptors and autophagy. Front. Immunol..

[68] Cho J.H., Lee H.J., Ko H.J., Yoon B.I., Choe J., Kim K.C., Hahn T.W., Han J.A., Choi S.S., Jung Y.M. (2017). The TLR7 agonist imiquimod induces anti-cancer effects via autophagic cell death and enhances anti-tumoral and systemic immunity during radiotherapy for melanoma. Oncotarget.

[69] Lee H.J., Kang S.J., Woo Y., Hahn T.W., Ko H.J., Jung Y.J. (2020). TLR7 Stimulation with Imiquimod Induces Selective Autophagy and Controls *Mycobacterium tuberculosis* Growth in Mouse Macrophages. Front. Microbiol..

[70] Periyasamy K.M., Ranganathan U.D., Tripathy S.P., Bethunaickan R. (2020). Vitamin D—A host directed autophagy mediated therapy for tuberculosis. Mol. Immunol..

[71] Mily A., Rekha R.S., Kamal S.M., Akhtar E., Sarker P., Rahim Z., Gudmundsson G.H., Agerberth B., Raqib R. (2013). Oral intake of phenylbutyrate with or without vitamin D3 upregulates the cathelicidin LL-37 in human macrophages: A dose finding study for treatment of tuberculosis. BMC Pulm. Med..

[72] Rekha R.S., Rao Muvva S.S., Wan M., Raqib R., Bergman P., Brighenti S., Gudmundsson G.H., Agerberth B. (2015). Phenylbutyrate induces LL-37-dependent autophagy and intracellular killing of *Mycobacterium tuberculosis* in human macrophages. Autophagy.

[73] Rao Muvva J., Ahmed S., Rekha R.S., Kalsum S., Groenheit R., Schon T., Agerberth B., Bergman P., Brighenti S. (2021). Immunomodulatory Agents Combat Multidrug-Resistant Tuberculosis by Improving Antimicrobial Immunity. J. Infect. Dis..

[74] Kim J.J., Lee H.M., Shin D.M., Kim W., Yuk J.M., Jin H.S., Lee S.H., Cha G.H., Kim J.M., Lee Z.W. (2012). Host cell autophagy activated by antibiotics is required for their effective antimycobacterial drug action. Cell Host Microbe.

[75] Krug S., Gupta M., Kumar P., Feller L., Ihms E.A., Kang B.G., Srikrishna G., Dawson T.M., Dawson V.L., Bishai W.R. (2023). Inhibition of host PARP1 contributes to the anti-inflammatory and antitubercular activity of pyrazinamide. Nat. Commun..

[76] Schiebler M., Brown K., Hegyi K., Newton S.M., Renna M., Hepburn L., Klapholz C., Coulter S., Obregon-Henao A., Henao Tamayo M. (2015). Functional drug screening reveals anticonvulsants as enhancers of mTOR-independent autophagic killing of *Mycobacterium tuberculosis* through inositol depletion. EMBO Mol. Med..

[77] Lim Y.J., Choi J.A., Choi H.H., Cho S.N., Kim H.J., Jo E.K., Park J.K., Song C.H. (2011). Endoplasmic reticulum stress pathway-mediated apoptosis in macrophages contributes to the survival of *Mycobacterium tuberculosis*. PLoS ONE.

[78] Xu P., Tang J., He Z.G. (2022). Induction of Endoplasmic Reticulum Stress by CdhM Mediates Apoptosis of Macrophage During *Mycobacterium tuberculosis* Infection. Front. Cell Infect. Microbiol..

[79] Pennini M.E., Liu Y., Yang J., Croniger C.M., Boom W.H., Harding C.V. (2007). CCAAT/enhancer-binding protein beta and delta binding to CIITA promoters is associated with the inhibition of CIITA expression in response to *Mycobacterium tuberculosis* 19-kDa lipoprotein. J. Immunol..

[80] Gupta P.K., Chakraborty P., Kumar S., Singh P.K., Rajan M.G., Sainis K.B., Kulkarni S. (2016). G1-4A, a Polysaccharide from Tinospora cordifolia Inhibits the Survival of *Mycobacterium tuberculosis* by Modulating Host Immune Responses in TLR4 Dependent Manner. PLoS ONE.

[81] Alonzi T., Aiello A., Sali M., Delogu G., Villella V.R., Raia V., Nicastri E., Piacentini M., Goletti D. (2024). Multiple antimicrobial and immune-modulating activities of cysteamine in infectious diseases. Biomed. Pharmacother..

[82] Boland R., Heemskerk M.T., Forn-Cuni G., Korbee C.J., Walburg K.V., Esselink J.J., Carvalho Dos Santos C., de Waal A.M., van der Hoeven D.C.M., van der Sar E. (2023). Repurposing Tamoxifen as Potential Host-Directed Therapeutic for Tuberculosis. mBio.

[83] Singhal A., Jie L., Kumar P., Hong G.S., Leow M.K., Paleja B., Tsenova L., Kurepina N., Chen J., Zolezzi F. (2014). Metformin as adjunct antituberculosis therapy. Sci. Transl. Med..

[84] Lachmandas E., Eckold C., Bohme J., Koeken V., Marzuki M.B., Blok B., Arts R.J.W., Chen J., Teng K.W.W., Ratter J. (2019). Metformin Alters Human Host Responses to *Mycobacterium tuberculosis* in Healthy Subjects. J. Infect. Dis..

[85] Bohme J., Martinez N., Li S., Lee A., Marzuki M., Tizazu A.M., Ackart D., Frenkel J.H., Todd A., Lachmandas E. (2020). Metformin enhances anti-mycobacterial responses by educating CD8+ T-cell immunometabolic circuits. Nat. Commun..

[86] Safe I.P., Lacerda M.V.G., Printes V.S., Praia Marins A.F., Rebelo Rabelo A.L., Costa A.A., Tavares M.A., Jesus J.S., Souza A.B., Beraldi-Magalhaes F. (2020). Safety and efficacy of N-acetylcysteine in hospitalized patients with HIV-associated tuberculosis: An open-label, randomized, phase II trial (RIPENACTB Study). PLoS ONE.

[87] Mannick J.B., Del Giudice G., Lattanzi M., Valiante N.M., Praestgaard J., Huang B., Lonetto M.A., Maecker H.T., Kovarik J., Carson S. (2014). mTOR inhibition improves immune function in the elderly. Sci. Transl. Med..

[88] Raien A., Davis S., Zhang M., Zitser D., Lin M., Pitcher G., Bhalodia K., Subbian S., Venketaraman V. (2023). Effects of Everolimus in Modulating the Host Immune Responses against *Mycobacterium tuberculosis* Infection. Cells.

[89] de Almeida P.E., Pereira de Sousa N.M., Rampinelli P.G., Silva R.V.S., Correa J.R., D’Avila H. (2023). Lipid droplets as multifunctional organelles related to the mechanism of evasion during mycobacterial infection. Front. Cell Infect. Microbiol..

[90] Gabor K.A., Fessler M.B. (2017). Roles of the Mevalonate Pathway and Cholesterol Trafficking in Pulmonary Host Defense. Curr. Mol. Pharmacol..

[91] Adewole O.O., Omotoso B.A., Ogunsina M., Aminu A., Ayoola O., Adedeji T., Awopeju O.F., Sogaolu O.M., Adewole T.O., Odeyemi A.O. (2023). Atorvastatin improves sputum conversion and chest X-ray severity score. Int. J. Tuberc. Lung Dis..

[92] Yang J., Zhang L., Qiao W., Luo Y. (2023). *Mycobacterium tuberculosis*: Pathogenesis and therapeutic targets. MedComm.

[93] Babunovic G.H., DeJesus M.A., Bosch B., Chase M.R., Barbier T., Dickey A.K., Bryson B.D., Rock J.M., Fortune S.M. (2022). CRISPR Interference Reveals That All-Trans-Retinoic Acid Promotes Macrophage Control of *Mycobacterium tuberculosis* by Limiting Bacterial Access to Cholesterol and Propionyl Coenzyme A. mBio.

[94] Stan D., Enciu A.M., Mateescu A.L., Ion A.C., Brezeanu A.C., Stan D., Tanase C. (2021). Natural Compounds with Antimicrobial and Antiviral Effect and Nanocarriers Used for Their Transportation. Front. Pharmacol..

[95] Nasim N., Sandeep I.S., Mohanty S. (2022). Plant-derived natural products for drug discovery: Current approaches and prospects. Nucleus.

[96] Agarwal A., Prasad R., Jain A. (2010). Effect of green tea extract (catechins) in reducing oxidative stress seen in patients of pulmonary tuberculosis on DOTS Cat I regimen. Phytomedicine.

[97] Butov D., Zaitseva S., Butova T., Stepanenko G., Pogorelova O., Zhelezniakova N. (2016). Efficacy and safety of quercetin and polyvinylpyrrolidone in treatment of patients with newly diagnosed destructive pulmonary tuberculosis in comparison with standard antimycobacterial therapy. Int. J. Mycobacteriol..

[98] Talebi A., Soltani R., Khorvash F., Jouabadi S.M. (2023). The Effectiveness of Silymarin in the Prevention of Anti-tuberculosis Drug-induced Hepatotoxicity: A Randomized Controlled Clinical Trial. Int. J. Prev. Med..

[99] Luangchosiri C., Thakkinstian A., Chitphuk S., Stitchantrakul W., Petraksa S., Sobhonslidsuk A. (2015). A double-blinded randomized controlled trial of silymarin for the prevention of antituberculosis drug-induced liver injury. BMC Complement. Altern. Med..

[100] Lara-Espinosa J.V., Arce-Aceves M.F., Lopez-Torres M.O., Lozano-Ordaz V., Mata-Espinosa D., Barrios-Payan J., Silva-Islas C.A., Maldonado P.D., Marquina-Castillo B., Hernandez-Pando R. (2022). Effect of Curcumin in Experimental Pulmonary Tuberculosis: Antimycobacterial Activity in the Lungs and Anti-Inflammatory Effect in the Brain. Int. J. Mol. Sci..

[101] Gupta P.K., Jahagirdar P., Tripathi D., Devarajan P.V., Kulkarni S. (2023). Macrophage targeted polymeric curcumin nanoparticles limit intracellular survival of *Mycobacterium tuberculosis* through induction of autophagy and augment anti-TB activity of isoniazid in RAW 264.7 macrophages. Front. Immunol..

[102] Tousif S., Singh D.K., Mukherjee S., Ahmad S., Arya R., Nanda R., Ranganathan A., Bhattacharyya M., Van Kaer L., Kar S.K. (2017). Nanoparticle-Formulated Curcumin Prevents Posttherapeutic Disease Reactivation and Reinfection with *Mycobacterium tuberculosis* following Isoniazid Therapy. Front. Immunol..

[103] Li Y., Luo W.W., Cheng X., Xiang H.R., He B., Zhang Q.Z., Peng W.X. (2022). Curcumin attenuates isoniazid-induced hepatotoxicity by upregulating the SIRT1/PGC-1alpha/NRF1 pathway. J. Appl. Toxicol..

[104] Yang H., Hu J., Chen Y.J., Ge B. (2019). Role of Sirt1 in innate immune mechanisms against *Mycobacterium tuberculosis* via the inhibition of TAK1 activation. Arch. Biochem. Biophys..

[105] Singh D.K., Tousif S., Bhaskar A., Devi A., Negi K., Moitra B., Ranganathan A., Dwivedi V.P., Das G. (2021). Luteolin as a potential host-directed immunotherapy adjunct to isoniazid treatment of tuberculosis. PLoS Pathog..

[106] Dwivedi V.P., Bhattacharya D., Yadav V., Singh D.K., Kumar S., Singh M., Ojha D., Ranganathan A., Van Kaer L., Chattopadhyay D. (2017). The Phytochemical Bergenin Enhances T Helper 1 Responses and Anti-Mycobacterial Immunity by Activating the MAP Kinase Pathway in Macrophages. Front. Cell Infect. Microbiol..

[107] Kumar S., Sharma C., Kaushik S.R., Kulshreshtha A., Chaturvedi S., Nanda R.K., Bhaskar A., Chattopadhyay D., Das G., Dwivedi V.P. (2019). The phytochemical bergenin as an adjunct immunotherapy for tuberculosis in mice. J. Biol. Chem..

[108] Bhaskar A., Kumari A., Singh M., Kumar S., Kumar S., Dabla A., Chaturvedi S., Yadav V., Chattopadhyay D., Prakash Dwivedi V. (2020). [6]-Gingerol exhibits potent anti-mycobacterial and immunomodulatory activity against tuberculosis. Int. Immunopharmacol..

[109] Patel N., Jagannath K., Vora A., Patel M., Patel A. (2017). A Randomized, Controlled, Phase III Clinical Trial to Evaluate the Efficacy and Tolerability of Risorine with Conventional Rifampicin in the Treatment of Newly Diagnosed Pulmonary Tuberculosis Patients. J. Assoc. Physicians India.

[110] Ayman M., Mahmoud M.C.G.a.A.S.S. (2014). Berberine Attenuates Isoniazid-lnduced Hepatotoxicity by Modulating Peroxisome Proliferator-Activated Receptor y, Oxidative Stress and Inflammation. Int. J. Pharmacol..

[111] Pahuja I., Negi K., Kumari A., Agarwal M., Mukhopadhyay S., Mathew B., Chaturvedi S., Maras J.S., Bhaskar A., Dwivedi V.P. (2023). Berberine governs NOTCH3/AKT signaling to enrich lung-resident memory T cells during tuberculosis. PLoS Pathog..

[112] Ozturk M., Chia J.E., Hazra R., Saqib M., Maine R.A., Guler R., Suzuki H., Mishra B.B., Brombacher F., Parihar S.P. (2021). Evaluation of Berberine as an Adjunct to TB Treatment. Front. Immunol..

[113] Dwivedi V.P., Bhattacharya D., Singh M., Bhaskar A., Kumar S., Fatima S., Sobia P., Kaer L.V., Das G. (2019). Allicin enhances antimicrobial activity of macrophages during *Mycobacterium tuberculosis* infection. J. Ethnopharmacol..

[114] Anand P.K., Kaul D., Sharma M. (2006). Green tea polyphenol inhibits *Mycobacterium tuberculosis* survival within human macrophages. Int. J. Biochem. Cell Biol..

[115] Sharma A., Vaghasiya K., Ray E., Gupta P., Gupta U.D., Singh A.K., Verma R.K. (2020). Targeted Pulmonary Delivery of the Green Tea Polyphenol Epigallocatechin Gallate Controls the Growth of *Mycobacterium tuberculosis* by Enhancing the Autophagy and Suppressing Bacterial Burden. ACS Biomater. Sci. Eng..

[116] Chen M., Deng J., Li W., Lin D., Su C., Wang M., Li X., Abuaku B.K., Tan H., Wen S.W. (2015). Impact of tea drinking upon tuberculosis: A neglected issue. BMC Public Health.

[117] Soh A.Z., Pan A., Chee C.B.E., Wang Y.T., Yuan J.M., Koh W.P. (2017). Tea Drinking and Its Association with Active Tuberculosis Incidence among Middle-Aged and Elderly Adults: The Singapore Chinese Health Study. Nutrients.

[118] Bai X., Oberley-Deegan R.E., Bai A., Ovrutsky A.R., Kinney W.H., Weaver M., Zhang G., Honda J.R., Chan E.D. (2016). Curcumin enhances human macrophage control of *Mycobacterium tuberculosis* infection. Respirology.

[119] Jahagirdar P.S., Gupta P.K., Kulkarni S.P., Devarajan P.V. (2020). Intramacrophage Delivery of Dual Drug Loaded Nanoparticles for Effective Clearance of *Mycobacterium tuberculosis*. J. Pharm. Sci..

[120] Yang H., Chen J., Chen Y., Jiang Y., Ge B., Hong L. (2020). Sirtuin inhibits M. tuberculosis -induced apoptosis in macrophage through glycogen synthase kinase-3beta. Arch. Biochem. Biophys..

[121] Yang H., Chen J., Chen Y., Jiang Y., Ge B., Hong L. (2021). Sirt1 activation negatively regulates overt apoptosis in Mtb-infected macrophage through Bax. Int. Immunopharmacol..

[122] Ahmad S., Bhattacharya D., Kar S., Ranganathan A., Van Kaer L., Das G. (2019). Curcumin Nanoparticles Enhance Mycobacterium bovis BCG Vaccine Efficacy by Modulating Host Immune Responses. Infect. Immun..

[123] Mukhopadhyay S., Pahuja I., Okieh A.A., Pandey D., Yadav V., Bhaskar A., Dwivedi V.P. (2024). Bergenin potentiates BCG efficacy by enriching mycobacteria-specific adaptive memory responses via the Akt-Foxo-Stat4 axis. Tuberculosis.

[124] Sharma S., Kalia N.P., Suden P., Chauhan P.S., Kumar M., Ram A.B., Khajuria A., Bani S., Khan I.A. (2014). Protective efficacy of piperine against *Mycobacterium tuberculosis*. Tuberculosis.

[125] Vora A., Patel S., Patel K. (2016). Role of Risorine in the Treatment of Drug—Susceptible Pulmonary Tuberculosis: A Pilot Study. J. Assoc. Physicians India.

[126] Rodriguez-Flores E.M., Mata-Espinosa D., Barrios-Payan J., Marquina-Castillo B., Castanon-Arreola M., Hernandez-Pando R. (2019). A significant therapeutic effect of silymarin administered alone, or in combination with chemotherapy, in experimental pulmonary tuberculosis caused by drug-sensitive or drug-resistant strains: In vitro and in vivo studies. PLoS ONE.

[127] Li M., Wu Z., Niu W., Wan Y., Zhang L., Shi G., Xi X. (2014). The protective effect of curcumin against the 19-kDa *Mycobacterium tuberculosis* protein-induced inflammation and apoptosis in human macrophages. Mol. Med. Rep..

[128] Hasan N., Yusuf N., Toossi Z., Islam N. (2006). Suppression of *Mycobacterium tuberculosis* induced reactive oxygen species (ROS) and TNF-alpha mRNA expression in human monocytes by allicin. FEBS Lett..

[129] Butova T., Zaitseva S., Butov D., Stepanenko G. (2016). Morphological changes in experimental tuberculosis resulting from treatment with quercetin and polyvinylpyrrolidone. Int. J. Mycobacteriol..

[130] Zhang Y., Qu X., Gao H., Zhai J., Tao L., Sun J., Song Y., Zhang J. (2020). Quercetin attenuates NLRP3 inflammasome activation and apoptosis to protect INH-induced liver injury via regulating SIRT1 pathway. Int. Immunopharmacol..

[131] Sanjay S., Girish C., Toi P.C., Bobby Z. (2021). Quercetin modulates NRF2 and NF-kappaB/TLR-4 pathways to protect against isoniazid- and rifampicin-induced hepatotoxicity in vivo. Can. J. Physiol. Pharmacol..

[132] Lee Y.Y., Tee V. (2023). Hepatoprotective effects of silymarin in management of liver injury caused by tuberculosis treatment. Drugs Context.

[133] Chen Q., Hu A., Ma A., Jiang F., Xiao Y., Chen Y., Huang R., Yang T., Zhou J. (2022). Effectiveness of Prophylactic Use of Hepatoprotectants for Tuberculosis Drug-Induced Liver Injury: A Population-Based Cohort Analysis Involving 6,743 Chinese Patients. Front. Pharmacol..

[134] Marjani M., Fahim F., Sadr M., Kazempour Dizaji M., Moniri A., Khabiri S., Tabarsi P., Velayati A.A. (2019). Evaluation of Silymarin for management of anti-tuberculosis drug induced liver injury: A randomized clinical trial. Gastroenterol. Hepatol. Bed Bench.

[135] Heo E., Kim D.K., Oh S.H., Lee J.K., Park J.H., Chung H.S. (2017). Effect of Prophylactic Use of Silymarin on Anti-tuberculosis Drugs Induced Hepatotoxicity. Tuberc. Respir. Dis..

[136] Marjani M., Baghaei P., Kazempour Dizaji M., Gorji Bayani P., Fahimi F., Tabarsi P., Velayati A.A. (2016). Evaluation of Hepatoprotective Effect of Silymarin Among Under Treatment Tuberculosis Patients: A Randomized Clinical Trial. Iran. J. Pharm. Res..

[137] Zhang S., Pan H., Peng X., Lu H., Fan H., Zheng X., Xu G., Wang M., Wang J. (2016). Preventive use of a hepatoprotectant against anti-tuberculosis drug-induced liver injury: A randomized controlled trial. J. Gastroenterol. Hepatol..

[138] Younes M., Aggett P., Aguilar F., Crebelli R., Dusemund B., Filipič M., Frutos M.J., Galtier P., Gott D., EFSA Panel on Food Additives and Nutrient Sources added to Food (ANS) (2018). Scientific opinion on the safety of green tea catechins. EFSA J..

[139] Hu J., Webster D., Cao J., Shao A. (2018). The safety of green tea and green tea extract consumption in adults—Results of a systematic review. Regul. Toxicol. Pharmacol..

[140] Ouyang J., Zhu K., Liu Z., Huang J. (2020). Prooxidant Effects of Epigallocatechin-3-Gallate in Health Benefits and Potential Adverse Effect. Oxid. Med. Cell Longev..

[141] Bellmann-Strobl J., Paul F., Wuerfel J., Dorr J., Infante-Duarte C., Heidrich E., Kortgen B., Brandt A., Pfuller C., Radbruch H. (2021). Epigallocatechin Gallate in Relapsing-Remitting Multiple Sclerosis: A Randomized, Placebo-Controlled Trial. Neurol. Neuroimmunol. Neuroinflamm..

[142] Chatree S., Sitticharoon C., Maikaew P., Pongwattanapakin K., Keadkraichaiwat I., Churintaraphan M., Sripong C., Sririwichitchai R., Tapechum S. (2021). Epigallocatechin gallate decreases plasma triglyceride, blood pressure, and serum kisspeptin in obese human subjects. Exp. Biol. Med..

[143] Rust R., Chien C., Scheel M., Brandt A.U., Dorr J., Wuerfel J., Klumbies K., Zimmermann H., Lorenz M., Wernecke K.D. (2021). Epigallocatechin Gallate in Progressive MS: A Randomized, Placebo-Controlled Trial. Neurol. Neuroimmunol. Neuroinflamm..

[144] Yin X., Zhu W., Tang X., Yang G., Zhao X., Zhao K., Jiang L., Li X., Zhao H., Wang X. (2024). Phase I/II clinical trial of efficacy and safety of EGCG oxygen nebulization inhalation in the treatment of COVID-19 pneumonia patients with cancer. BMC Cancer.

[145] Andres S., Pevny S., Ziegenhagen R., Bakhiya N., Schafer B., Hirsch-Ernst K.I., Lampen A. (2018). Safety Aspects of the Use of Quercetin as a Dietary Supplement. Mol. Nutr. Food Res..

[146] Di Pierro F., Iqtadar S., Khan A., Ullah Mumtaz S., Masud Chaudhry M., Bertuccioli A., Derosa G., Maffioli P., Togni S., Riva A. (2021). Potential Clinical Benefits of Quercetin in the Early Stage of COVID-19: Results of a Second, Pilot, Randomized, Controlled and Open-Label Clinical Trial. Int. J. Gen. Med..

[147] Han M.K., Barreto T.A., Martinez F.J., Comstock A.T., Sajjan U.S. (2020). Randomised clinical trial to determine the safety of quercetin supplementation in patients with chronic obstructive pulmonary disease. BMJ Open Respir. Res..

[148] Kozhukhov S., Parkhomenko A., Lutay Y., Dovganych N., Study I. (2024). Impact of quercetin in patients with myocardial infarction. A multicenter, randomized, and open-label pilot study. Hellenic J. Cardiol..

[149] Yang K., Chen J., Zhang T., Yuan X., Ge A., Wang S., Xu H., Zeng L., Ge J. (2022). Efficacy and safety of dietary polyphenol supplementation in the treatment of non-alcoholic fatty liver disease: A systematic review and meta-analysis. Front. Immunol..

[150] Flaig T.W., Gustafson D.L., Su L.J., Zirrolli J.A., Crighton F., Harrison G.S., Pierson A.S., Agarwal R., Glode L.M. (2007). A phase I and pharmacokinetic study of silybin-phytosome in prostate cancer patients. Investig. New Drugs.

[151] Aryan H., Farahani R.H., Chamanara M., Elyasi S., Jaafari M.R., Haddad M., Sani A.T., Ardalan M.A., Mosaed R. (2022). Evaluation of the efficacy of oral nano-silymarin formulation in hospitalized patients with COVID-19: A double-blind placebo-controlled clinical trial. Phytother. Res..

[152] Sharifi-Rad J., Rayess Y.E., Rizk A.A., Sadaka C., Zgheib R., Zam W., Sestito S., Rapposelli S., Neffe-Skocinska K., Zielinska D. (2020). Turmeric and Its Major Compound Curcumin on Health: Bioactive Effects and Safety Profiles for Food, Pharmaceutical, Biotechnological and Medicinal Applications. Front. Pharmacol..

[153] Cheng A.L., Hsu C.H., Lin J.K., Hsu M.M., Ho Y.F., Shen T.S., Ko J.Y., Lin J.T., Lin B.R., Ming-Shiang W. (2001). Phase I clinical trial of curcumin, a chemopreventive agent, in patients with high-risk or pre-malignant lesions. Anticancer. Res..

[154] Doostkam A., Iravani K., Malekmakan L., Gholamabbas G., Roozbeh J., Soltaniesmaeili A. (2024). The effectiveness of curcumin as a safe agent on hearing threshold improvement in patients with chronic kidney disease: A double-blind, placebo-controlled trial. Sci. Rep..

[155] Noppakun K., Jitraknatee J., Suteeka Y., Ruengorn C., Nochaiwong S., Gunaparn S., Phrommintikul A., Wongcharoen W. (2024). Effect of Curcuminoids on Contrast-Induced Acute Kidney Injury after Elective Coronary Angiography or Intervention: A Pilot Randomized, Double-Blind, Placebo-Controlled Study. Cardiorenal Med..

[156] Soltani M., Hosseinzadeh-Attar M.J., Rezaei M., Alipoor E., Vasheghani-Farahani A., Yaseri M., Rezayat S.M. (2024). Effect of nano-curcumin supplementation on cardiometabolic risk factors, physical and psychological quality of life, and depression in patients with coronary slow flow phenomenon: A randomized double-blind clinical trial. Trials.

[157] Yaikwawong M., Jansarikit L., Jirawatnotai S., Chuengsamarn S. (2024). Curcumin extract improves beta cell functions in obese patients with type 2 diabetes: A randomized controlled trial. Nutr. J..

[158] Yaikwawong M., Jansarikit L., Jirawatnotai S., Chuengsamarn S. (2024). The Effect of Curcumin on Reducing Atherogenic Risks in Obese Patients with Type 2 Diabetes: A Randomized Controlled Trial. Nutrients.

[159] Garcia-Martinez B.I., Ruiz-Ramos M., Pedraza-Chaverri J., Santiago-Osorio E., Mendoza-Nunez V.M. (2023). Effect of Resveratrol on Markers of Oxidative Stress and Sirtuin 1 in Elderly Adults with Type 2 Diabetes. Int. J. Mol. Sci..

[160] Mahjabeen W., Khan D.A., Mirza S.A. (2022). Role of resveratrol supplementation in regulation of glucose hemostasis, inflammation and oxidative stress in patients with diabetes mellitus type 2: A randomized, placebo-controlled trial. Complement. Ther. Med..

[161] Shaito A., Posadino A.M., Younes N., Hasan H., Halabi S., Alhababi D., Al-Mohannadi A., Abdel-Rahman W.M., Eid A.H., Nasrallah G.K. (2020). Potential Adverse Effects of Resveratrol: A Literature Review. Int. J. Mol. Sci..

[162] Zhou Y., Zeng Y., Pan Z., Jin Y., Li Q., Pang J., Wang X., Chen Y., Yang Y., Ling W. (2023). A Randomized Trial on Resveratrol Supplement Affecting Lipid Profile and Other Metabolic Markers in Subjects with Dyslipidemia. Nutrients.

[163] Popat R., Plesner T., Davies F., Cook G., Cook M., Elliott P., Jacobson E., Gumbleton T., Oakervee H., Cavenagh J. (2013). A phase 2 study of SRT501 (resveratrol) with bortezomib for patients with relapsed and or refractory multiple myeloma. Br. J. Haematol..

[164] Mankowski R.T., You L., Buford T.W., Leeuwenburgh C., Manini T.M., Schneider S., Qiu P., Anton S.D. (2020). Higher dose of resveratrol elevated cardiovascular disease risk biomarker levels in overweight older adults—A pilot study. Exp. Gerontol..

[165] Li J., Zhang C., Xu Y., Yang L. (2024). Efficacy and safety of berberine plus 5-ASA for ulcerative colitis: A systematic review and meta-analysis. PLoS ONE.

[166] Ming J., Yu X., Xu X., Wang L., Ding C., Wang Z., Xie X., Li S., Yang W., Luo S. (2021). Effectiveness and safety of Bifidobacterium and berberine in human hyperglycemia and their regulatory effect on the gut microbiota: A multi-center, double-blind, randomized, parallel-controlled study. Genome Med..

[167] Panigrahi A., Mohanty S. (2023). Efficacy and safety of HIMABERB(R) Berberine on glycemic control in patients with prediabetes: Double-blind, placebo-controlled, and randomized pilot trial. BMC Endocr. Disord..

[168] Rondanelli M., Gasparri C., Petrangolini G., Allegrini P., Avenoso D., Fazia T., Bernardinelli L., Peroni G., Patelli Z., Mansueto F. (2023). Berberine phospholipid exerts a positive effect on the glycemic profile of overweight subjects with impaired fasting blood glucose (IFG): A randomized double-blind placebo-controlled clinical trial. Eur. Rev. Med. Pharmacol. Sci..

[169] Zhao J.V., Yeung W.F., Chan Y.H., Vackova D., Leung J.Y.Y., Ip D.K.M., Zhao J., Ho W.K., Tse H.F., Schooling C.M. (2021). Effect of Berberine on Cardiovascular Disease Risk Factors: A Mechanistic Randomized Controlled Trial. Nutrients.

[170] Bandala C., Carro-Rodriguez J., Cardenas-Rodriguez N., Pena-Montero I., Gomez-Lopez M., Hernandez-Roldan A.P., Huerta-Cruz J.C., Munoz-Gonzalez F., Ignacio-Mejia I., Dominguez B. (2024). Comparative Effects of Gymnema sylvestre and Berberine on Adipokines, Body Composition, and Metabolic Parameters in Obese Patients: A Randomized Study. Nutrients.

[171] Cui Y., Zhou Q., Jin M., Jiang S., Shang P., Dong X., Li L. (2024). Research progress on pharmacological effects and bioavailability of berberine. Naunyn Schmiedebergs Arch. Pharmacol..

[172] Harrison S.A., Gunn N., Neff G.W., Kohli A., Liu L., Flyer A., Goldkind L., Di Bisceglie A.M. (2021). A phase 2, proof of concept, randomised controlled trial of berberine ursodeoxycholate in patients with presumed non-alcoholic steatohepatitis and type 2 diabetes. Nat. Commun..

[173] Qiao M., Lei C., Tan C., Lu C., Chen Z., Zhang Q., Wang Z. (2023). Efficacy and safety of berberine for premature ventricular contractions: A meta-analysis and systematic review of randomized controlled trials. Pharm. Biol..

[174] Di Stadio A., D’Ascanio L., Vaira L.A., Cantone E., De Luca P., Cingolani C., Motta G., De Riu G., Vitelli F., Spriano G. (2022). Ultramicronized Palmitoylethanolamide and Luteolin Supplement Combined with Olfactory Training to Treat Post-COVID-19 Olfactory Impairment: A Multi-Center Double-Blinded Randomized Placebo-Controlled Clinical Trial. Curr. Neuropharmacol..

[175] Ntalouka F., Tsirivakou A. (2023). Luteolin: A promising natural agent in management of pain in chronic conditions. Front. Pain Res..

[176] Preisler H.D., Raza A., Larson R., Browman G., Goldberg J., Grunwald H., Vogler R., Bennett J., Gottlieb A., D’Arrigo P. (1986). Limited efficacy of a four-day course of high-dose cytosine arabinoside in the treatment of poor-risk patients with acute nonlymphocytic leukemia. Cancer Chemother. Pharmacol..

[177] Terzo S., Amato A., Magan-Fernandez A., Castellino G., Calvi P., Chianetta R., Giglio R.V., Patti A.M., Nikolic D., Firenze A. (2023). A Nutraceutical Containing Chlorogenic Acid and Luteolin Improves Cardiometabolic Parameters in Subjects with Pre-Obesity: A 6-Month Randomized, Double-Blind, Placebo-Controlled Study. Nutrients.

[178] Crichton M., Marshall S., Isenring E., Lohning A., McCarthy A.L., Molassiotis A., Bird R., Shannon C., Koh A., McPherson I. (2024). Effect of a Standardized Ginger Root Powder Regimen on Chemotherapy-Induced Nausea and Vomiting: A Multicenter, Double-Blind, Placebo-Controlled Randomized Trial. J. Acad. Nutr. Diet..

[179] Crichton M., Marshall S., Marx W., Isenring E., Vazquez-Campos X., Dawson S.L., Lohning A. (2023). Effect of Ginger Root Powder on Gastrointestinal Bacteria Composition, Gastrointestinal Symptoms, Mental Health, Fatigue, and Quality of Life: A Double-Blind Placebo-Controlled Trial. J. Nutr..

[180] Ivashkin V.T., Kudryavtseva A.V., Krasnov G.S., Poluektov Y.M., Morozova M.A., Shifrin O.S., Beniashvili A.G., Mamieva Z.A., Kovaleva A.L., Ulyanin A.I. (2022). Efficacy and safety of a food supplement with standardized menthol, limonene, and gingerol content in patients with irritable bowel syndrome: A double-blind, randomized, placebo-controlled trial. PLoS ONE.

[181] Dludla P.V., Cirilli I., Marcheggiani F., Silvestri S., Orlando P., Muvhulawa N., Moetlediwa M.T., Nkambule B.B., Mazibuko-Mbeje S.E., Hlengwa N. (2023). Bioactive Properties, Bioavailability Profiles, and Clinical Evidence of the Potential Benefits of Black Pepper (*Piper nigrum*) and Red Pepper (*Capsicum annum*) against Diverse Metabolic Complications. Molecules.

[182] Ziegenhagen R., Heimberg K., Lampen A., Hirsch-Ernst K.I. (2021). Safety Aspects of the Use of Isolated Piperine Ingested as a Bolus. Foods.

[183] Salimo Z.M., Yakubu M.N., da Silva E.L., de Almeida A.C.G., Chaves Y.O., Costa E.V., da Silva F.M.A., Tavares J.F., Monteiro W.M., de Melo G.C. (2023). Chemistry and Pharmacology of Bergenin or Its Derivatives: A Promising Molecule. Biomolecules.

[184] Rana S.V., Pal R., Vaiphei K., Sharma S.K., Ola R.P. (2011). Garlic in health and disease. Nutr. Res. Rev..

[185] Deng Y., Ho C.T., Lan Y., Xiao J., Lu M. (2023). Bioavailability, Health Benefits, and Delivery Systems of Allicin: A Review. J. Agric. Food Chem..

[186] Mehmood S., Maqsood M., Mahtab N., Khan M.I., Sahar A., Zaib S., Gul S. (2022). Epigallocatechin gallate: Phytochemistry, bioavailability, utilization challenges, and strategies. J. Food Biochem..

[187] Aghababaei F., Hadidi M. (2023). Recent Advances in Potential Health Benefits of Quercetin. Pharmaceuticals.

[188] Carrillo-Martinez E.J., Flores-Hernandez F.Y., Salazar-Montes A.M., Nario-Chaidez H.F., Hernandez-Ortega L.D. (2024). Quercetin, a Flavonoid with Great Pharmacological Capacity. Molecules.

[189] Madaan R., Singla R.K., Kumar S., Dubey A.K., Kumar D., Sharma P., Bala R., Singla S., Shen B. (2022). Bergenin—A Biologically Active Scaffold: Nanotechnological Perspectives. Curr. Top. Med. Chem..

[190] Shen L., Liu C.C., An C.Y., Ji H.F. (2016). How does curcumin work with poor bioavailability? Clues from experimental and theoretical studies. Sci. Rep..

[191] Yucel C., Karatoprak G.S., Acikara O.B., Akkol E.K., Barak T.H., Sobarzo-Sanchez E., Aschner M., Shirooie S. (2022). Immunomodulatory and anti-inflammatory therapeutic potential of gingerols and their nanoformulations. Front. Pharmacol..

[192] Zhang W., Zheng Q., Song M., Xiao J., Cao Y., Huang Q., Ho C.T., Lu M. (2021). A review on the bioavailability, bio-efficacies and novel delivery systems for piperine. Food Funct..

[193] Zhang Z., Li X., Sang S., McClements D.J., Chen L., Long J., Jiao A., Wang J., Jin Z., Qiu C. (2022). A review of nanostructured delivery systems for the encapsulation, protection, and delivery of silymarin: An emerging nutraceutical. Food Res. Int..

[194] Baldwin P.R., Reeves A.Z., Powell K.R., Napier R.J., Swimm A.I., Sun A., Giesler K., Bommarius B., Shinnick T.M., Snyder J.P. (2015). Monocarbonyl analogs of curcumin inhibit growth of antibiotic sensitive and resistant strains of *Mycobacterium tuberculosis*. Eur. J. Med. Chem..

[195] Hsu C.Y., Pallathadka H., Gupta J., Ma H., Al-Shukri H.H.K., Kareem A.K., Zwamel A.H., Mustafa Y.F. (2024). Berberine and berberine nanoformulations in cancer therapy: Focusing on lung cancer. Phytother. Res..

[196] Saini N., Chopra B., Dhingra A.K. (2023). Synergistic Effect of Piperine and its Derivatives: A Comprehensive Review. Curr. Drug Res. Rev..

